# Insect Antifeedant Benzofurans from *Pericallis* Species

**DOI:** 10.3390/molecules28030975

**Published:** 2023-01-18

**Authors:** Carmen E. Díaz, Braulio M. Fraga, Adriana G. Portero, Iván Brito, Carmen López-Balboa, Liliana Ruiz-Vásquez, Azucena González-Coloma

**Affiliations:** 1 Instituto de Productos Naturales y Agrobiología, Consejo Superior de Investigaciones Científicas (CSIC), Avda. Astrofísico F. Sánchez 3, 38206 La Laguna, Tenerife, Spain; 2 Departamento de Química, Universidad de Antofagasta, Antofagasta 124000, Chile; 3 Instituto de Ciencias Agrarias, Consejo Superior de Investigaciones Científicas (CSIC), Serrano 115 dpdo., 28006 Madrid, Spain; 4 Laboratorio de Productos Naturales Antiparasitarios de la Amazonia, Centro de Investigación de Recursos Naturales, Universidad Nacional de la Amazonia Peruana (UNAP), 16001 Iquitos, Peru

**Keywords:** *Pericallis*, aerial parts, transformed roots, benzofurans, biotransformation, antifeedant, and post-ingestive activity

## Abstract

In this work, we have studied the benzofurans of *Pericallis echinata* (aerial parts and transformed roots), *P. steetzii* (aerial parts and transformed roots), *P. lanata* (aerial parts), and *P. murrayi* (aerial parts and roots). This work has permitted the isolation of the new benzofurans 10-ethoxy-11-hydroxy-10,11-dihydroeuparin (**10**), (-)-eupachinin A ethyl ether (**12**), 11,15-didehydro-eupachinin A (**13**), 10,12-dihydroxy-11-angelyloxy-10,11-dihydroeuparin (**14**), 2,4-dihydroxy-5-formyl-acetophenone (**15**) isolated for the first time as a natural product, 11-angelyloxy-10,11-dihydroeuparin (**16**), and 12-angelyloxyeuparone (**17**), along with several known ones (**1**–**9, 11**). In addition, the incubation of the abundant component, 6-hydroxytremetone (**1**), with the fungus *Mucor plumbeus* has been studied. Benzofurans in the tremetone series (**1**, **1a**, **2**–**5**, **18**, **18a**), the euparin series (**6**, **7**, **7a**, **8**–**10**, **14**, **16**), and the eupachinin-type (**11**, **12**) were tested for antifeedant effects against the insect *Spodoptera littoralis*. The antifeedant compounds (**1**, **4**, **6**, **11**, **12**) were further tested for postingestive effects on *S. littoralis* larvae. The most antifeedant compounds were among the tremetone series, with 3-ethoxy-hydroxy-tremetone (**4**) being the strongest antifeedant. Glucosylation of **1** by its biotransformation with *Mucor plumbeus* gave inactive products. Among the euparin series, the dihydroxyangelate **14** was the most active, followed by euparin (**6**). The eupachinin-type compounds (**11**, **12**) were both antifeedants. Compounds **4, 11,** and **12** showed antifeedant effects without postingestive toxicity to orally dosed *S. littoralis* larvae. Euparin (**6)** had postingestive toxicity that was enhanced by the synergist piperonyl butoxide.

## 1. Introduction

*Pericallis*, a genus of the tribe Senecioneae (subtribe Senecioninae) in the Asteraceae family, comprises seventeen taxa of woody and herbaceous species which are endemic to Canary Islands, Madeira, and Azores [1]. The name *Pericallis* was introduced by Don in 1834 for *P. tussilaginis* [2] but was not used until 134 years later when *Pericallis* was considered close to the African *Cineraria* and several *Cineraria* and *Senecio* species were included in it [3]. However, a study on the molecular phylogeny of *Pericallis* found it closer to the American *Packera* genus [4]. Later, the monophyly of *Pericallis* was strongly supported and, again, a close relation with the African *Cineraria* genus was suggested considering morphologic and molecular ITS data [5]. More recently, the influence of geographical isolation, multiple habitat shifts, and hybridization in the evolution of this genus has been studied, using more taxon’s sampling and studying both nuclear and chloroplast genomes’ data [1,6].

*Senecio* and *Pericallis* species produce pyrrolizidine alkaloids (PAs) [7]. PAs are considered feeding deterrents for insect herbivores. A number of insect species from different taxa have evolved adaptations to sequester, store, and utilize plant PAs against their predators and parasitoids [8,9]. 

Ethanolic extracts of some PA-producing plants from the Canary Islands, including *Pericallis* (*P. appendiculata*, *P. echinata*, *P. hansenii*, *P. multiflora*, *P. steetzii*), were overall more active insect antifeedants than the alkaloidal ones. Considering that the alkaloidal fraction of these ethanolic extracts accounted for a maximum of 2%, the authors concluded that the chemistry of the non-alkaloidal fraction could explain most of their antifeedant effects [7]. Non-alkaloidal fractions of *Senecio* (and former *Senecio* such as the Canarian endemism *Bethencourtia*) species contain insect antifeedant sesquiterpenoids with different skeletons [10,11,12,13,14]. However, little is known on the non-alkaloidal defensive chemistry of *Pericallis* spp.

In this work, we have studied the benzofuran-based defensive chemistry of *Pericallis echinata* (L. fil.) B. Nord. (aerial parts and transformed roots), *P. steetzii* (Bolle) B. Nord. (aerial parts and transformed roots), *P. lanata* (L’Hér.) B. Nord. (aerial parts), and *P. murrayi* (Bornm.) B. Nord. (aerial parts and roots). Our study led to the isolation of the known benzofurans (**1**–**9, 11**), the new ones 10-ethoxy-11-hydroxy-10,11-dihydroeuparin (**10**), (-)-eupachinin A ethyl ether (**12**), 11,15-didehydro-eupachinin A (**13**), 10,12-dihydroxy-11-angelyloxy-10,11-dihydroeuparin (**14**), 11-angelyloxy-10,11-dihydroeuparin (**16**), 12-angelyloxyeuparone (**17**), and 2,4-dihydroxy-5-formyl-acetophenone (**15**) isolated for the first time as a natural product. In addition, the incubation of the abundant component, 6-hydroxytremetone (**1**), with the fungus *Mucor plumbeus* has been studied. Moreover, an improved identification of euparone and tremetone derivatives has been carried out using NMR data, allowing the correction of the ^13^C data of some of these compounds. Benzofurans in the tremetone series (**1**, **1a**, **2**–**5**, **18**, **18a**), the euparin series (**6**, **7**, **7a**, **8**–**10**, **14**, **16**), and the eupachinin-type (**11**, **12**) were tested for antifeedant effects against the insect *Spodoptera littoralis*. The antifeedant compounds (**1**, **4**, **6**, **11**, **12**) were further tested for postingestive effects on *S. littoralis* larvae.

## 2. Results and Discussion

### 2.1. Components of Pericallis

**Figure 1:**
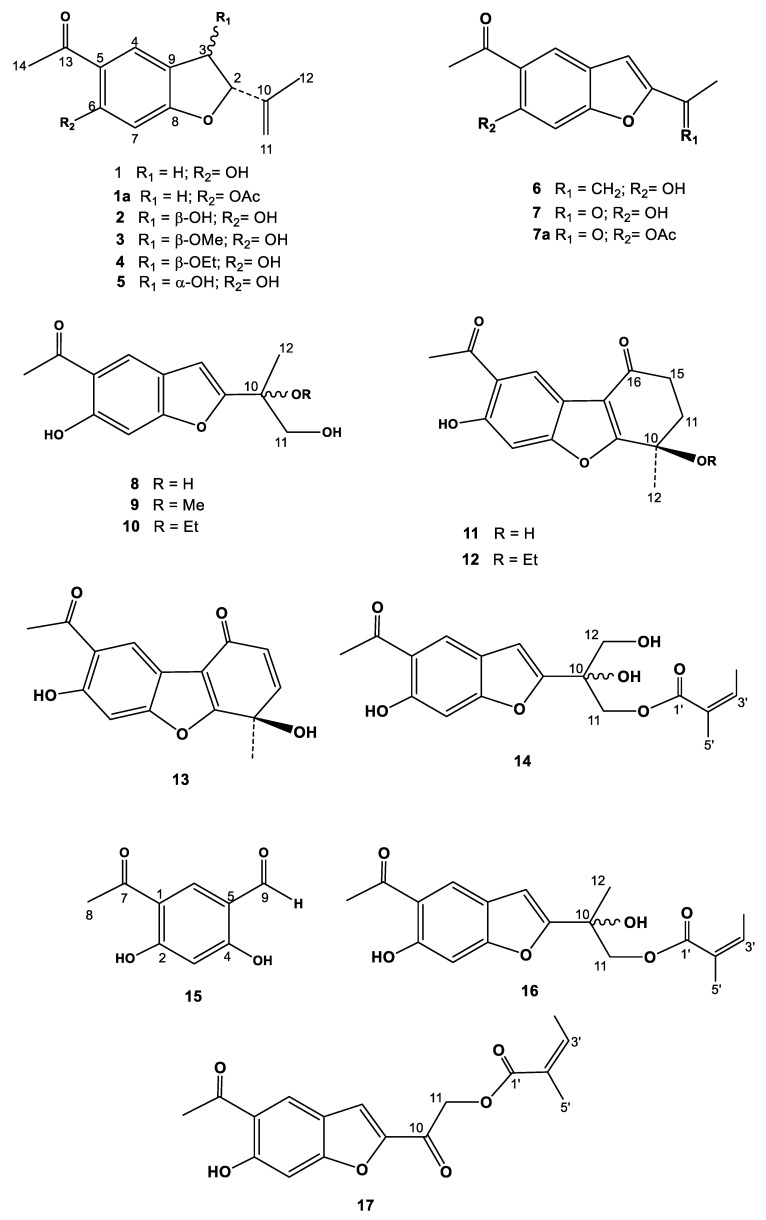
Chemical structures of compounds **1–17**.

All the species studied contained benzofurans (Figure 1) as the major components, along with triterpenoids and sterols. Most benzofuran-type sesquiterpenes have been isolated from Asteraceae species [15], with a few examples from *Senecio* [11], but none have been reported from *Pericallis*.

The benzofurans 6-hydroxytremetone (**1**) [16,17], 3β,6-dihydroxytremetone (**2**) [18,19], 3β-methoxy-6-hydroxytremetone (**3**) [20,21,22], 3β-ethoxy-6-hydroxytremetone (**4**) [23], 3α,6-dihydroxytremetone (**5**) [24], euparin (**6**) [22,25,26], euparone (**7**) [22,27], 10,11-dihydroxy-10,11-dihydroeuparin (**8**) [28], 10-methoxy-11-hydroxy-10,11-dihydroeuparin (**9**) [29], 10-ethoxy-11-hydroxy-10,11-dihydroeuparin (**10**), eupachinin A (**11**) [30], eupachinin A ethyl ether (**12**), 11,15-didehydro eupachinin A (**13**), 10,12-dihydroxy-11-angelyloxy-10,11-dihydroeuparin (**14**), 2,4-dihydroxy-5-formyl-acetophenone (**15**) [31], 11-angelyloxy-10,11-dihydroeuparin (**16**), and 12-angelyloxyeuparone (**17**) were isolated from the different *Pericallis* species and plant parts. The pentacyclic triterpenes 3β,16β-dihydroxy-lup-20(29)-ene [32], 11-oxo-α-amirin [33], 11-oxo-β-amirin [34], 3α,16β-dihydroxy-urs-12-ene [35], 3β,16β-dihydroxy-urs-12-ene [35], and 3β,16β-dihydroxy-olean-12-ene [36], and the sterols campesterol, stigmasterol, and β-sitosterol, were also identified in these species.

We describe now for the first time the ^13^C NMR (Table 1) spectra of 3β,6-dihydroxytremetone (**2**) [18,19], 3β-ethoxy-6-hydroxytremetone (**4**) [23], 3α,6-dihydroxytremetone (**5**) [24], 10,11-dihydroxy-10,11-dihydroeuparin (**8**) [28], and 10-methoxy-11-hydroxy-10,11-dihydroeuparin (**9**) [29]. In addition, we have corrected some assignments in the carbon resonances of 6-hydroxytremetone (**1**) [17] and euparin (**6**) [26]. The ^13^C NMR spectrum of euparone (**7**), reported by Hussein (1992) [28], does not match with our data and those described by Habtemariam (2001) [22], which has also now been reassigned.

To complete these studies, derivatives of 6-hydroxytremetone (**1**) and euparone (**7**) were prepared. Compounds **1** and **7** were acetylated to give 6-AcO derivatives **1a** and **7a**, respectively, and their NMR and MS spectroscopic data confirmed the presence of the AcO group at C-6.

The high-resolution mass spectrum (HREIMS) of compound **11** showed the molecular ion at *m/z* 274.0834 (C_15_H_14_O_5_). In its ^1^H NMR spectrum were observed signals corresponding to the ring A of a 6-hydroxy-5-acetyl benzofuran grouping. Other resonances present in it were a methyl geminal to an oxygenated function at δ 1.78 and two methylene groups located in contiguous positions. The hydrogens of one of them resonate at δ 2.63 (1H, ddd, J = 17.0, 6.5 and 5.0 Hz, H-15β) and 2.92 (1H, ddd, J = 17.0, 9.0 and 5.0 Hz, H-15α), while that those of the second methylene, coupled with the previous one, appear centred at δ 2.36 (m, 2H-11). In its ^13^C NMR spectrum were detected signals of two methyls, two methylenes, two aromatic protons, and two carbonyl groups, one the acetyl group on C-5 to δ 194.1 and another of an α,β-unsaturated carbonyl group at δ 204.6 (C-16). Moreover, a quaternary carbon bearing an oxygen atom at δ 67.4 (C-10) and other six unsaturated quaternary carbons also appear in this spectrum.

**Figure 2:**
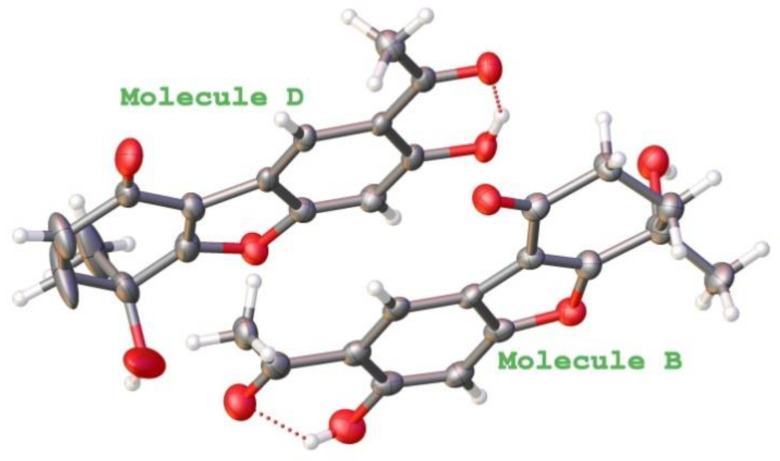
View of the eupachinin (B) and didehydro-eupachinin (D) molecules. Molecules A and C were omitted for sake.

The location of the different functional groups was established based on the connectivity observed in the HSQC and HMBC spectra. The correlations in the HMBC experiment of the aromatic protons and the phenolic proton, with their respective geminal and neighbourhood carbons, confirmed that the ring A of the molecule was similar to that of other benzofurans of euparin type. This spectrum also indicated that the two methylene groups were located between the carbonyl group and the quaternary carbon bearing an oxygen atom. Thus, connectivities of H-11 with C-2, C-10, and C-15, of the two H-15 with C-10, C-11, and C-16, and of methyl group (H-12) with C-2, C-10, and C-11 were observed. These spectroscopic data allowed us to assign to our product the structure **11** [37]. To confirm this hypothesis, we submit it to an X-ray diffraction analysis (Figure 2).

The asymmetric unit of the title cocrystal, 3C_15_H_14_O_5_—C_15_H_12_O_5_ contains four structural units. The A-C molecules with the same structure, C_15_H_14_O_5_, and the D-molecule with an additional 11,15-double bond, C_15_H_12_O_5_. Intramolecular O—H···O interactions occur in all four independent molecules with graph-set motif S(6). In the crystal, only B molecule is linked to C and D molecules by O—H···O intermolecular hydrogen bonds and π-π stacking interactions between the benzene rings A and C molecules (centroid–centroid distances ranging from 3.696 (3) to 3.721 (4) Å). The conformation of the molecules A and B are similar showing an envelope conformation for the methyl-cyclohexanone ring. The C molecule has a screw-boat conformation for the methyl-cyclohexanone ring, and the methyl group is in an axial position. The molecule D has a planar conformation (mean torsion angle 5.106 (12)° due to the presence of the 11,15-double bond.

Once the relative structure of compound **11** and its 11,15-didehydro derivative **13** had been established by X-ray analysis, a pair of enantiomers (-) and (+)-eupachinin A were isolated from *Eupatorium chinense* with [α]_D_ -5.1 and +4.8, respectively [30]. Spectroscopic data were identical to those of our compound of optical rotation −8.33, which was consequently identified as (-)-eupachinin A (**11**). The presence of the undescribed 11,15-didehydro derivative **13** in the crystal fraction of the X-ray analysis was unexpected, because NMR signals of the 11,15-double bond were not observed in the ^1^H and ^13^C spectra of the fractions studied.

(-)-10-Ethoxy-eupachinin A (**12**), the corresponding ethyl derivative of **11**, was also isolated from *P. echinata*. With the molecular formula C_17_H_18_O_5_, its ^1^H and ^13^C NMR spectra were very similar to that of compound **11**, except that now the signals corresponding to a 10-ethoxy group also appear. This compound probably is an artefact formed in the extraction of the plant with ethanol. The same applies to the undescribed benzofuran 10-ethoxy-11-hydroxy-euparin (**10**), also obtained from aerial part of *P. echinata* together with its known analogues **8** and **9**.

The new benzofuran **14** was isolated from the transformed roots of *P. echinata* and *P.steetzzi*. Its high-resolution mass spectrum showed the molecular ion at *m/z* 348.1211 (C_18_H_20_O_7_). The ^1^H NMR spectrum displayed the signals of the aromatic protons H-3, H-4, and H-7 at δ 6.75, 6.99, and 7.95, respectively, the phenolic proton associated with the carbonyl group at δ 12.44 and the acetyl group at δ 2.69 (H-14), which are similar to those observed in other euparin derivatives. The main differences with euparin (**6**) were the disappearance of the methylene double bond and the presence in **14** of singlets corresponding to two hydroxyls and also the characteristic signals of an angelyloxy group. The oxygenated methylenes, H-11 and H-12, resonate as pairs of doublets at δ 4.55 and 4.61 (J = 11.6 Hz) and δ 3.90 and 3.98 (J = 12.7 Hz), respectively, while the signals of the angelate group appear at δ 1.83 *(t*, H-5′), 1.91 (*dd*, H-4′), and 6.12 (*ddd*, H-3′). In its ^13^C NMR spectrum (Table 2), eighteen signals were observed, three methyls, two methylenes, four methines, and nine quaternary carbons. The HMBC experiment showed correlations in the angelyloxy group of the H-4′ and H-5′ methyls with C-1′/C-2′/C-3′, and the location of this ester at C-11, with crosspeaks of H-11 with C-2/C-10/C-12/C-1′, H-12 with C-2 and OH-12 with C-2/C-10/C-12. Thus, the structure 10, 12-dihydroxy-11-angelyloxy-10,11-dihydroeuparin (**14**) was assigned to this new product.

Compound **15** (benzofuran precursor), also isolated from the aerial part of *P. echinata*, was determined as 2,4-dihydroxy-5-formyl-acetophenone (5-acetyl-2,4-dihydroxy-benzaldehyde) (**15**). The HRMS showed the molecular ion at *m/z* 180.0428 in accordance with the formula C_9_H_8_O_4_. In its ^1^H NMR spectrum only singlets were observed, the methyl ketone at δ 2.64 and two aromatic protons at δ 6.46 and 7.99 due to H-6 and H-3, respectively, while the aldehyde hydrogen resonated at δ 9.77. Two phenolic protons were displaced at low-field, δ 11.62 and 13.08, due to hydrogen bonds with carbonyl groups, which were assigned to HO-4 and HO-2, respectively, considering the connectivities observed in the HMBC experiment. The ^13^C NMR spectrum, described in the experiment, displayed the corresponding signals of carbons bearing hydrogens and those of four substituted aromatic carbons. The position of the substituents was established based on the correlations observed in the HMBC spectrum: H-3 with C-1/C-4; H-6 with C-2/C-4/C-7/C-9; H-8/with C-1/C-7; and H-9 with C-4/C-5. This compound **15** had been obtained as an intermediate in the synthesis of neobavachalcone [31] and now has been isolated for the first time as a natural product.

The new benzofurans 11-angelyloxy-10,11-dihydroeuparin (**16**) and 12-angelyloxyeuparone **(17**) were isolated from the transformed roots of *P. steetzii.* Compound **16** was isolated as an oil. Its HRMS was in accordance with the formula C_18_H_20_O_5_. The ^1^H NMR spectrum showed the characteristic signals of a benzofuran ring, which were similar to those observed in euparin (**6**), but now the isopropylene group of this molecule has been substituted in **16** by a -CH(CH_3_)-CH_2_OAng group. Thus, the two H-11 protons of the oxymethylene group resonates as a pair of double doublets at δ 4.33 and 4.40, with 12.0 and 7.0 Hz coupling, respectively, while the H-12 methyl and the H-10 appears at δ 1.41 (*d*, J = 7.0 Hz) and 3.32 (*m*). In the ^13^C NMR spectrum the corresponding carbons were detected at δ 66.2 (C-11), 15.5 (C-12) and 33.3 (C-10). The typical signals of the angelate group in both spectra were similar to those described for **14**. Therefore, the structure of this compound was determined as 11-angelyloxy-10,11-dihydroeuparin (**16**). This compound has not been previously described in the chemical literature.

The HRMS of 12-Angelyloxyeuparone (**17**) was in accordance with the structural formula C_17_H_16_O_6_. The ^1^H NMR spectrum showed singlet resonances of H-3, H-4, and H-7 at 7.57, 8.20, and 7.10, which were similar to those observed for euparone (**7**) at δ 7.50, 8.23, and 7.10, respectively. The same occurs with the methyl group at δ 2.74 (H-14) and the associated proton of the hydroxyl group at δ 12.57, in comparison with 2.70 and 12.63 described for **7** [27]. Moreover, in **17** the oxymethylene group appears as a singlet at δ 5.34, and the signals of the angelate ester were similar to those observed in compounds **14** and **16**. The presence of this angelate ester was confirmed in the EIMS with fragments at *m/z* 217 and 203, which were originated from the molecular ion by cleavage of the C-10, C-11 bond and loss of the angelyloxy group, respectively.

### 2.2. Biotransformation of 6-Hydroxytremetone (1)

**Figure 3:**
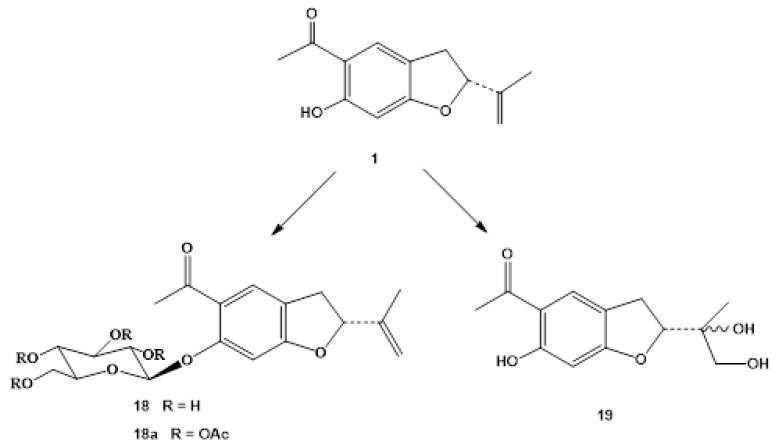
Biotransformation compounds of **1** by *Mucor plumbeus*.

Two benzofuran derivatives, 6-hydroxytremetone β-D-glucoside (**18**) and 6,10,11-trihydroxytremetone (**19**), have been obtained from the biotransformation of 6-hydroxytremetone (**1**) with *Mucor plumbeus* (Figure 3). This fungus has a broad specificity of substrate and has been used in the biotransformation of sesquiterpenes and diterpenes [38,39,40].

The major biotransformation compound was 6-hydroxytremetone β-D-glucoside (**18**). Its high-resolution mass spectrum showed the molecular ion at *m/z* 380, which corresponds to the molecular formula C_19_H_24_O_8_. The ^1^H NMR spectrum was similar to that of the substrate, but with additional signals of a glucose moiety, with proton signal between δ 3.44 and 5.05. In the HMBC spectrum, a correlation between the anomeric proton H-1`and C-6 was observed. The coupling constant of H-1′ (J = 7.3 Hz) indicated an axial β-configuration for this substituent, which was confirmed by the resonance of the anomeric carbon at δ 103.4 [41]. Acetylation of this compound led to the acetyl derivative **18a**, which showed the molecular ion at 548.1874 *m/z*, corresponding to the molecular formula C_27_H_32_O_12_. Their NMR spectra confirmed this structure.

The second product, structure **19,** was obtained in very low yield. Its mass spectrum showed the molecular ion at *m/z*: 252.1008 (C_13_H_16_O_5_), which indicates the introduction of two new oxygen atoms into the molecule with respect to the substrate **1**. In addition to the signals observed in the starting benzofuran, an AB system appears, at δ 3.55 and 3.77 due to the two methylene protons (H-11) and the signal of a methyl at δ 1.12, which was assigned as a substituent on C-2, based on the correlations observed in the HMBC experiment. In the ^13^C NMR spectrum (Table 3), the disappearance of the signals of the vinyl carbons of the substrate and the presence of two new resonances at δ 67.0 and 73.5 ppm, were typical of C-10 and C-11 respectively, which are linked to oxygens. Previously, this product had been isolated from *Helianthopsis stuebelii* [42]. It could be originated by opening of the corresponding epoxide during the isolation procedure, as also occurred in the formation of 10,11-dihydroxyeuparin (**8**).

### 2.3. Antifeedant and Postingestive Effects

Table 4 shows the antifeedant and postingestive effects of the tested compounds. The most antifeedant compounds were among the tremetone series (**1**, **1a**, **2–5**, **18**, **18a**), with 3-ethoxy-hydroxy-tremetone (**4**) being the strongest antifeedant (1st in EC_50_ ranking), followed by 6-hydroxy-tremetone (**1**) (2nd in EC_50_ ranking) and **1a** (6th in EC_50_ ranking). Glucosylation of **1** by its biotransformation with *Mucor plumbeus* gave inactive products (**18** and **18a**). Among the euparin series (**6**, **7**, **7a**, **8–10**, **14**, **16**), the dihydroxyangelate **14** (third in the EC_50_ ranking) was the most active, followed by euparin (**6**) and euparin angelate (**16**) (sixth and seventh in the EC_50_ ranking). The eupachinin-type compounds (**11**, **12**) were both antifeedants (fourth and fifth in the EC_50_ ranking) and this is the first report on the antifeedant effects of this type of benzofurans.

The antifeedant compounds (**1**, **4**, **6**, **11**, **12**) were further tested for postingestive effects on *S. littoralis* larvae (Table 4). At a dose of 40 µg/larvae, **4, 11,** and **12** showed antifeedant effects without postingestive toxicity (pANCOVA2 > 0.05). Compound **6** (euparin) had postingestive toxicity at 40 µg/larvae (pANCOVA2 = 0.035) that was lost at 20 µg/larvae, except when the larvae were pre-treated with PBO (piperonyl butoxide, a synergist), resulting in a significant antifeedant postingestive effect and indicating the involvement of a PSMO (polysubstrate monoxygenases) detoxification mechanism for **6** in *S. littoralis.*

Insect antifeedant effects of benzofurans have been previously described. Natural dihydrobenzofurans such as remirol and aurones were antifeedants to *Spodoptera litura* [43,44,45]. Euparin (**6**) was antifeedant to *S. litura,* but methyleuparin showed stronger effects [43]. Inclusion of an acetyl or methoxy on the aromatic group of benzofurans gave effective antifeedant activity, while hydroxylation or methylation decreased this effect [43]. In this work, ethoxylation of the C3 hydroxy group in the furan ring gave the strongest antifeedant effect (**4**), while acetylation of the aromatic hydroxyl group reduced or eliminated the antifeedant activity in the tremetone series (**1** vs. **1a**). Modifications of the side chain also affected the activity. For example, the oxidation of euparin **6** in C10 eliminated the activity (in **7** and **7a**), and the presence of a C11 angelate substituent in the side chain (**16**, **14**) increased the activity (**8**)**.**

Previously, methyleuparin showed a moderate growth inhibition effect on *Peridroma saucia* [45], and 6-hydroxy tremetone derivatives and euparin affected the ratio of *Tenebrio molitor* larval pupation [46]. In this work, we have shown growth inhibitory effects on *S. littoralis* larvae for the tremetone derivative **4** and eupachinin compounds **11** and **12,** while euparin **6**, with an unsaturated benzofuran ring, was the most toxic compound. The oxidation of the unsaturated furan ring turns benzofurans in alkylating agents, becoming cytotoxic and mutagenic [47]. This will explain the larval postingestive toxicity of euparin (**6**), with an unsaturated furan ring. The larval toxicity of **6** was enhanced by the application of the synergist PBO. PBO is a specific inhibitor of microsomal oxidases and resistance-associated esterases [48] and has shown insecticidal synergistic effects with synthetic unsaturated benzofurans [49], indicating that insects can detoxify these compounds as shown here.

## 3. Materials and Methods

### 3.1. General Procedure

Melting points were determined with a Reichert Thermovar apparatus and are uncorrected (Reichert Technologies, Buffalo, NY, USA). Optical rotations were measured at room temperature on a Perkin Elmer 343 polarimeter (Perkin Elmer, Waltham, MA, USA)**.** IR spectra were recorded with a Perkin-Elmer 1600 FT spectrometer (Perkin Elmer, Waltham, MA, USA). ^1^H and ^13^C NMR spectra were run in CDCl_3_ solution at 500.1 and 125.8 MHz, respectively, in a Bruker AMX-500 spectrometer (Bruker Corporation, Billerica, MA, USA) with pulsed field gradient, using this solvent signal (CDCl3, at δH7.26 and δC77.0) as internal standard. Chemical shifts are given in ppm (δ). EI and HREIMS were taken in a Micromass Autospect instrument (Manchester, UK) at 70 eV (probe). HRESIMS data were recorded on a Waters Micromass LCT Premier XE (Manchester, UK). Column chromatographies were made on silica gel (40∓63 μm, Merck, Darmstadt, Germany), and Sephadex LH-20 (Amersham Pharmacia Biotech AB, Uppsala, Sweden). Preparative and semipreparative HPLC was performed on a Beckman System Gold 125P (Beckman Coulter Life Sciences, Brea, CA, USA) equipped with a diode-array detector Beckman Coulter 168 and preparative Interstil Prep-sil (Gasukuro Kogio) (20 mm × 250 mm, 10 µm) and semipreparative Beckman Ultrasphere silica (10 mm × 250 mm, 5 µm) columns.

### 3.2. Plant Material

All *Pericallis* species were collected at their flowering stage (aerial parts) in the Canary Islands (Spain) and identified by Dr. Arnoldo Santos. All voucher specimens (ORT) have been deposited at the Herbarium del Jardín de Aclimatación de La Orotava, Tenerife, Spain.

*Pericallis echinata* (L. fil.) B. Nord. from Tenerife Island (Los Realejos, 28°20′59.99″ N 16°35′59.99″ W) was collected in March (aerial parts) and May (seeds) (ORT 32003). *P. steetzii* (Bolle) B. Nord from Gomera Island (Garajonay National Park, 28°07′34″ N 17°14′14″ W) was collected in May (aerial parts) and June (seeds) 1991 (ORT 33451). *P. murrayi* (Bornm.) B. Nord. from El Hierro Island (28°16′07″ N 16°36′20″ O) was collected in April 2009 (aerial parts and roots) (ORT 27594). *P. lanata* DC. from Tenerife Island (Barranco del Río, 28°34′10″ N 16°18′48″ O) was collected in May 1994 (aerial parts) (ORT 32001).

### 3.3. Plant Material

*Agrobacterium rhizogenes* ATCC-15834 was inoculated with a needle to the stem of aseptic plantlets germinated from seeds and cultured on agar medium containing 30 g/mL sucrose and half-strength MS medium [50]. The induced hairy roots were excised and cultured on hormone-free half-Gamborg B5 solid medium [51] supplemented with 30 g/L sucrose and 0.5 mg/mL ampicillin to eliminate bacteria. The axenic hairy roots obtained were subcultured every 25–30 days in the dark at 25 °C on the same solid medium without antibiotics. Then, they were cultivated in the dark at 25°C in 250 mL Erlenmeyer flasks containing 100 mL of half-GB liquid medium supplemented with 30 g/L sucrose and shaken on a rotary shaker at 90 rpm. After five weeks, the hairy roots were harvested and separated from the culture medium by filtration through filter paper under vacuum.

### 3.4. DNA Extraction and Analysis

Total genomic DNA was extracted from transformed root tissue and from the untransformed root plants by using a “GenElute^TM^ Plant Genomic DNA Miniprep Kit” (Sigma-Aldrich, St. Louis, MO, USA). Plasmid DNA from *A. rhizogenes* strain ATCC-15834 was used as a positive control. Polymerase chain reaction was performed using REDExtract-N-Amp Plant PCR Kit (Sigma) to detect the insertion of T_L_-DNA of *A. rhizogenes* ATCC-15834 in the transformed roots. The oligonucleotide primers for T_L_-DNA were 5′-ATGGATCCCAAATTGCTATTCCTTCCA-3′ and 5′- TTAGGCTTCTTTCTTCAGG TTTA-3′ which amplify a segment complementary to the 5′ coding sequence of *rol* B to the 3′ coding sequence of *rol* C on the T_L_-DNA region. PCR amplification was performed in a DNA thermal cycler (Applied Biosystems 2700, Foster City, CA) under the following conditions: initial denaturation at 94 °C for 2 min, followed by 30 cycles of 90 °C for 30 s, annealing at 55 °C for 1 min, with a final extension at 72 °C for 5 min. The PCR reaction mixture was electrophoresed on a 1.2% agarose gel using tris-acetate-EDTA buffer and visualized by ethidium bromide staining under ultraviolet light at 260 nm.

### 3.5. Extraction and Isolation of Compounds from Pericallis

*Pericallis* plant parts (aerial and roots) were dried in open air in the shade and grinded to give dry plant materials (*P. echinata*, Pe, 4.700 g; *P. lanata*, Pl, 980 g; *P. steetzii*, Ps, 1800 g; *P. murrayi* aerial, Pma, 1251.0 g; *P. murrayi* roots, Pmr, 687.5 g). Hairy roots were freeze-dried and powdered to give transformed roots dried materials (*P. echinata*, PeTR, 112 g and *P. steetzii*, PsTR, 49.7 g). The dry materials were exhaustively extracted with EtOH in a Soxhlet. The solvent was evaporated at reduced pressure to give crude extracts (Pe,120 g, PeTR 41 g, Pl 175 g, Ps 180 g, PsTR 11.4 g Pma 137 g, Pmr 90.2 g).

The ethanolic extracts were chromatographed with silica gel vacuum liquid chromatography columns (VLC column, 10 × 25 cm) eluted with a hexane-EtOAc-MeOH gradients. Further, the main fractions were chromatographed on Si gel columns, Sephadex LH-20 and/or preparative normal phase HPLC eluted with different solvents and proportions of hexane-EtOAc and/or CH_2_Cl_2_/MeOH.

The following compounds were isolated: 6-hydroxytremetone (**1**) (Pe 0.49%, Pma 0.09% of the extract), 3β,6-dihydroxytremetone (**2**) (Pe 0.003%, PeTR 0.0025% of the extract), 3β-methoxy-6-hydroxytremetone (**3**) (Pe 0.001%), 3β-ethoxy-6-hydroxytremetone (**4**) (Pe 0.59%, Pl 0.019%, Ps 0.002%, Pma 0.01%, Pmr 0.001%, PeTR 0.005%, PsTR 0.02% of the extract), 3α,6-dihydroxytremetone (**5**) (Pe 0.001% of the extract), euparin (**6**) (Pe 0.135%, Pl 0.22%, Ps 0.04%, Pma 0.08%, Pmr 0.35%, PeTR 0.17%, PsTR 0.2% of the extract), euparone (**7**) (Pe 0.08%, Pma 0.02%, Pmr 0.03%, PeTR 0.02%, PsTR 0.045% of the extract), 10,11-dihydroxy-10,11-dihydroeuparin (**8**) (Pe 0.003% of the extract), 10-methoxy-11-hydroxy-10,11-dihydroeuparin (**9**) (Pe 0.001% of the extract), 10-ethoxy-11-hydroxy-10,11-dihydroeuparin (**10**) (Pe 0.01% of the extract), (-)-eupachinin A (**11**) (Pe 0.003% of the extract), (-)-eupachinin A ethyl ether (**12**) (Pe 0.004% of the extract), 10,12-dihydroxy-11-angelyloxy-10,11-dihydroeuparin (**14**) (PeTR 0.00025%, PsTR 0.0043%), 2,4-dihydroxy-5-formyl-acetophenone (**15**) (Pe 0.005% of the extract), 11-angelyloxy-10,11-dihydroeuparin (**16**) (PsTR 0.07% of the extract), 12-angelyloxyeuparone (**17**) (PsTR 0.02% of the extract).

### 3.6. Benzofurans

#### 3.6.1. 6-Hydroxytremetone (1)

White crystals, m.p. 48–50 °C; [α]_D_^20^ -42.2 (c 0.62, EtOH); IR (KBr) λ_max_: 2540, 1640, 1485, 1255, 1135, 1040 cm^−1^;^1^H NMR (500 MHz): δ 1.75 (3H, t, *J =* 1.4 Hz, H-12), 2.52 (3H, s, H-14), 2.96 (1H, dd, *J =*15.3, 7.5, 1.6 Hz, H-3α), 3.29 (1H, dd, *J =* 15.3, 9.5, 1.6 Hz, H-3β), 4.93 (1H, t, *J =* 1.4 Hz, H-11), 5.07 (1H, s ancho, H-11), 5.25 (1H, dt, *J =* 7.5, 9.5 Hz, H-2β), 6.35 (1H, s, H-7), 7.48 (1H, t, *J =* 1.6 Hz, H-4), 12.96 (1H, s, OH).; ^13^C NMR (125 MHz): See Table 1; EIMS *m/z* (rel. int.): 218 [M]+ (100), 203 (98), 175 (61), 161 (9), 160 (26), 157 (7), 132 (3), 115 (5); HREIMS *m/z*: 218.0942 [M]^+^. Calculated for C_13_H_14_O_3_, 218.0943.

#### 3.6.2. 3β,6-. Dihydroxytremetone (2)

Colourless oil; [α]_D_^20^ -13.6 (c 0.17, CHCl_3_); ^1^H NMR (500 MHz): δ 1.74 (3H, br s, H-12), 2.58 (3H, s, H-14), 4.95 (1H, t, J = 1.7 Hz, H-11), 4.97 (1H, d, J = 3.3 Hz, H-2β), 5.06 (1H, br s, H-11), 5.08 (1H, d, J = 3.3 Hz, H-3α), 6.44 (1H, s, H-7), 7.77 (1H, s, H-4), 12.99 (1H, s, H0-6); ^13^C NMR (125 MHz): See Table 1; EIMS *m/z* (rel. int.): 234 [M]^+^ (25), 216 (90), 201 (100), 191 (6), 178 (35), 149 (19), 115 (12); HREIMS *m/z*: 234.0891 [M]^+^. Calculated for C_13_H_14_O_4,_ 234.0892.

#### 3.6.3. 3β-. Methoxy-6-Hydroxytremetone (3)

Colourless oil; ^1^H NMR (500 MHz): δ 1.72 (3H, s, H-12), 2.58 (3H, s, H-14), 3.41 (3H, s, -OMe), 4.71 (1H, d, J = 2.5 Hz, H-3), 4.94 and 5.06 (each 1H, br s, H-11), 5.09 (1H, d, J = 2.5 Hz, H-2), 6.45 (1H, s, H-7), 7.75 (1H, s, H-4); ^13^C NMR (125 MHz): See Table 1; EIMS *m/z* (rel. int.): 248 [M]^+^ (100), 232 (16), 217 (57), 203 (20), 201 (16), 157 (15), 129 (9). HREIMS *m/z*: 248.1043 [M]^+^. Calculated for C_14_H_16_O_3_, 248.1049.

#### 3.6.4. 3β-. Ethoxy-6-Hydroxytremetone (4)

White crystals, m.p. 71–73 °C; [α]_D_^20^–13.6 (c 0.17, CHCl_3_); ^1^H NMR (500 MHz): δ 1.25 (3H, t, *J* = 7.0 Hz, H-2′), 1.70 (3H, s, H-12), 2.56 (3H, s, H-14), 3.62 (2H, c, *J* = 7.0 Hz, H-1′), 4.77 (1H, d, *J* = 2.5 Hz, H-3), 4.93 and 5.06 (each 1H, s, H-11), 5.07 (1H, d, *J* = 2.5 Hz, H-2), 6.42 (1H, s, H-7), 7.72 (1H, s, H-4), 12.99 (1H, s, OH); ^13^C NMR (125 MHz): See Table 1; EIMS *m/z* (rel. int.): 262 [M]^+^ (100), 245 (8), 233 (12), 219 (3), 217 (49), 203 (16); HREIMS *m/z*: 262.1208 [M]^+^. Calculated for C_15_H_18_O_4,_ 262.1205.

#### 3.6.5. 3α,6-. Dihydroxytremetona (5)

Colourless oil; [α]_D_^20^–6.9 (c 0.13, CHCl_3_); ^1^H NMR (500 MHz): δ 1.91 (3H, br s, H-12), 2.59 (3H, s, H-14), 5.05 (1H, d, J = 6.1 Hz, H-2), 5.17 (1H, br s, H-3), 5.21 and 5.26 (each 1H, s, H-11), 6.45 (1H, s, H-7), 7.82 (1H, s, H-4), 12.99 (1H, s, HO-6); ^13^C NMR (125 MHz): See Table 1; HRESIMS (negative-ion mode) *m/z*: 233.0810 [M-H]^+^. Calculated for C_13_H_13_O_4,_ 233.0814.

#### 3.6.6. Euparin (6)

Yellow crystals, m.p. 132–134 °C; ^1^H NMR (500 MHz): δ 2.10 (3H, s, H-12), 2.67 (3H, s, H-14), 5.18 (1H, d, *J =*1.3 Hz, H-11), 5.75 (1H, s, H-11), 6.54 (1H, s, H-3), 6.97 (1H, s, H-7), 7.89 (1H, s, H-4), 12.49 (1H, s, OH); ^13^C NMR (125 MHz): See Table 1; EIMS *m/z* (rel. int.): 262 [M]^+^ (100), 245 (8), 233 (12), 219 (3), 217 (49), 203 (16); HREIMS *m/z*: 262.1208 [M]^+^. Calculated for C_15_H_18_O_4,_ 262.1208.

#### 3.6.7. Euparone (7)

Amorphous solid; ^1^H NMR (500 MHz): δ 2.59 (3H, s, H-11), 2.73 (3H, s, H-13), 7.09 (1H, s, H-7), 7.46 (1H, br s, H-3), 8.17 (1H, s, H-4), 12.54 (1H, s, OH); ^13^C NMR (125 MHz): See Table 1; EIMS *m/z* (rel. int.): 218 [M]^+^ (59), 203 (100), 204 (9), 165 (11), 137 (7), 107 (15); HREIMS *m/z*: 218.0579 [M]^+^. Calculated for C_12_H_10_O_4_, 218.0579.

#### 3.6.8. 10,11-. Dihydroxy-10,11-Dihydroeuparin (**8**)

Colourless oil; ^1^H NMR (500 MHz): δ 1.59 (3H, s, H-12), 2.68 (3H, s, H-14), 3.69, 4.00 (1H each signal, d, *J* = 11.0 Hz, H-11), 6.66 (1H, s, H-3), 6.98 (1H, s, H-7), 7.92 (1H, s, H-4), 12.43 (1H, s, -OH); ^13^C NMR (125 MHz): See Table 1; EIMS *m/z* (rel. int.): 250 [M]^+^ (10), 219 (100), 203 (10), 176 (3); HREIMS *m/z*: 250.0841 [M]^+^. Calculated for C_13_H_14_O_5,_ 250.0841.

#### 3.6.9. 10-Methoxy-11-Hydroxy-10,11-Dihydroeuparin (9)

Colourless oil; ^1^H NMR (500 MHz): δ 1.63 (3H, s, H-12), 2.69 (3H, s, H-14), 3.23 (3H, s, -OMe), 3.73 and 3.92 (each 1H, d, *J* = 11.0 Hz, H-11), 6.67 (1H, s, H-3), 7.02 (1H, s, H-7), 7.95 (1H, s, H-4), 12.44 (1H, s, -OH); EIMS *m/z* (rel. int.): 264 [M]^+^ (5), 234 (14), 233 (100), 218 (9), 203 (22); HREIMS *m/z*: 264.0999 [M]^+^. Calculated for C_14_H_16_O_5,_ 264.0998.

#### 3.6.10. 10-Ethoxy-11-Hydroxy-10,11-Dihydroeuparin (**10**)

Colourless oil; [α]_D_ =–3 (c, 0.13, CHCl_3_); ^1^H NMR (500 MHz): δ 1.18 (3H, t, *J* = 7.0 Hz, -OCH_2_CH_3_), 1.63 (3H, s, H-12), 2.68 (3H, s, H-14), 3.31 and 3.49 (each 1H, dd, *J* = 8.8, 7.0 Hz, -OCH_2_CH_3_), 3.71 and 3.89 (each 1H, d, *J =* 11.0 Hz, H-11), 6.64 (1H, br. s, H-3), 7.00 (1H, s, H-7), 7.93 (1H, s, H-4), 12.42 (1H, s, -OH); EIMS *m/z* (rel. int.): 278 [M]^+^ (11), 248 (14), 247 (86), 232 (19), 219 (100), 203 (49); HREIMS *m/z*: 278.1153 [M]^+^. Calculated for C_15_H_18_O_5_, 180.0423.

#### 3.6.11. (-)-. Eupachinin A (11)

White crystal (hexane/EtOAc); [α]_D_ = -8.33 (c, 0.36, CHCl_3_); IR (KBr) λ_max_: 2540, 1640, 1483, 1257, 1134, 1039 cm^−1^; ^1^H NMR (500 MHz) δ 1.78 (3H, s, H-14), 2.36 (2H, m, H-12), 2.63 (1H, ddd, *J* = 17.0, 6.6, 5.0Hz, H-11β), 2.74 (3H, s, H-16), 2.92 (1H, ddd, *J* = 17.0, 9.0, 5.0 Hz, H-11α), 7.05 (1H, s, H-7), 8.43 (1H, s, H-4), 12.61 (1H, s, OH); EIMS *m/z* (rel. int.): 274 [M]^+^ (56), 259 (100), 257 (13), 241 (4), 231 (12), 203 (11); HREIMS *m/z*: 274.0834 [M]^+^. Calculated for C_15_H_14_O_5,_ 274.0841.

#### 3.6.12. X-ray Crystallography Data of (-)-Eupachinin A (11) and (-)-Didehydroeupachinin A (13)

Colourless crystals, C_60_H_54_O_20_, Mr = 1095.03, triclinic space group P1, *a* = 7.4090 (5) Å, *b* = 12.9440(7) Å, *c* = 13.8110 (7) Å, α = 97.232(4)°, β= 102.730(5)°, γ = 94.243(5)°, V = 1274.57 (13) Å^3^, Z = 1, µ (Cu Kα) = 0.90 cm^−1^. Intensity data were collected at 293 °K on a Enraf-Nonius Kappa CCD diffractometer using Cu Kα radiation and a graphite monochromator. No. of measured, independent, and observed [I > 2σ(I)] reflections 19038, 9410, and 5198, respectively (R_int_ = 0.042). Refinement: R[F2 > 2σ(F2)] = 0.077, wR(F2) = 0.265, S = 1.03. No. of reflections = 9410, no. of parameters = 738, no. of restraints = 4. H-atom parameters constrained, (Δ/σ)_max_ = 0.780, Δρ_max_, = 0.36 e Å^−3^ and Δρ_min_ =−0.24 e Å^−3^. Computer programs: *SHELXL2013* (Sheldrick, 2013). Supplementary Data have been deposited with the Cambridge Crystallographic Data Centre, no. 2031183. Copies of available material can be obtained, free of charge, via http://www.ccdc.cam.ac.uk/conts/retrieving.html, accessed on 30 April 2021) or from the CCDC, 12 Union Road, Cambridge CB2 1EZ, UK (Fax: +44-1223-336033; e-mail.: deposit@ccdc.cam.ac.uk).

#### 3.6.13. (-)-. Eupachinin A Ethyl Ether (12)

Amorphous solid; [α]_D_ = −3.70 (c, 0.27, CHCl_3_); ^1^H NMR (500 MHz): δ 1.15 (3H, t, *J* = 7.0 Hz, -OCH_2_CH_3_), 1.71 (3H, s, H-14), 2.18 (1H, ddd, *J* = 13.7, 11.0, 4.5 Hz, H-12β), 2.45 (1H, dt, *J* = 13.7, 4.5 Hz, H-12α), 2.57 (1H, dt, *J* = 17.1, 4.8 Hz, H-11β), 2.74 (3H, s, H-16), 3.00 (1H, ddd, *J* = 17.1, 11.0, 4.5 Hz, H-11α), 3.37 and 3.64 (each 1H, dd, *J* = 8.7, 7.0 Hz, -OCH_2_CH_3_), 7.08 (1H, s, H-7), 8.48 (1H, s, H-4), 12.59 (1H, s, -OH); EIMS *m/z* (rel. int.): 302 [M]^+^ (52), 287 (25), 259 (52), 257 (100), 241 (16), 229 (8), 214 (8), 115 (8); HREIMS *m/z*: 302.1152 [M]^+^. Calculated for C_17_H_18_O_5_, 302.1154.

#### 3.6.14. 10,12-. Dihydroxy-11-Angelyloxy-10,11-Dihydroeuparin (14)

^1^H NMR (500 MHz): δ (3H, t, J = 1.4 Hz, H-4′), 1.91 (3H, dd, J = 7.4, 1.6 Hz, H-5′), 2.33 (1H, br. s, HO-10), 2.69 (3H, s, H-14), 3.52 (1H, s, HO-12), 3.90 and 3.98 (each 1H, br. d, *J =* 12.6 Hz, H-12), 4.55 and 4.61 (1H each, d, *J =* 11.6 Hz, H-11), 6.12 (1H, qd, J = 7.3, 1.4 Hz, H-3′), 6.75 (1H, d, *J =* 0.9 Hz, H-3), 6.99 (1H, br. s, H-7), 7.95 (1H, s, H-4), 12.44 (1H, s, HO-6); EIMS *m/z* (rel. int.) 348 [M]^+^ (6), 235 (22), 230 (24), 217 (11), 215 (20), 203 (15), 189 (17), 83 (100); HREIMS *m/z*: 348.1211 [M]^+^. Calculated for C_18_H_20_O_7_, 348.1209).

#### 3.6.15. 2,4-. Dihydroxy-5-Formyl-Acetophenone (15)

Amorphous solid; ^1^H NMR (500 MHz): δ 2.64 (3H, s, H-8), 6.46 (1H, s, H-6), 7.99 (1H, s, H-3), 9.77 (1H, s, -CHO), 11.62 (1H, s, HO-4), 13.08 (1H, s, HO-2); ^13^C NMR (125 MHz): δ 114.8 (C-1), 169.7 (C-2), 104.9 (C-3), 167.8 (C-4), 114.5 (C-5), 139.6 (C-6), 202.4 (C-7), 26.2 (C-8), 194.0 (C-9); EIMS *m/z* (rel. int.): 180 [M]^+^ (60), 165 (100), 123 (6), 109 (4); HREIMS *m/z*: 180.0428 [M]^+^. Calculated for C_9_H_8_O_4,_ 180.0423.

#### 3.6.16. 11-Angelyloxy-10,11-Dihydroeuparin (16)

^1^H NMR (CDCl_3_, 500 MHz): δ 1.41 (3H, d, J = 7.0 Hz, H-12), 1.85 (3H, t, J = 1.5 Hz, H-5′), 1.92 (3H, dd, J = 7.2, 1.5 Hz, H-4′), 2.68 (3H, s, H-14), 3.32 (1H, m, H-10), 4.33 and 4.40 (each 1H, dd, J = 12.0, 7.0 Hz, H-11), 6.06 (1H, qd, J = 7.3, 1.3 Hz, H-3′), 6.40 (1H, s, H-3), 6.96 (1H, s, H-7), 7.88 (1H, s, H-4), 12.43 (1H, s, -OH); EIMS *m/z*, (rel. int.): 316 [M]^+^ (4), 217 (40), 216 (100), 201 (53), 198 (5), 185 (7), 173 (6), 83 (51); HREIMS *m/z*: 316.1304 [M]^+^. Calculated for C_18_H_20_O_5,_ 316.1310.

#### 3.6.17. 12-Angelyloxyeuparone (17)

^1^H NMR (CDCl_3_, 200 MHz): δ 2.01 (3H, br s, H-5′), 2.05 (3H, d, J = 7.2 Hz, H-4′), 2.74 (3H, s, H-13), 5.34 (2H, s, H-12), 6.20 (1H, m, H-3′), 7.10 (1H, s, H-7), 7.57 (1H, s, H-3), 8.20 (1H, s, H-4), 12.57 (1H, s, -OH); EIMS *m/z* (rel. int.): 316 [M]^+^ (46), 234 (9), 217 (4), 203 (73), 189 (3), 83 (100), 55 (60); HREIMS *m/z*: 316.0958 [M]^+^. Calculated for C_17_H_16_O_6,_ 316.0946.

### 3.7. Acetylation of 6-Hydroxytremetone (1)

Compound **1** (10 mg) in pyridine (0.5 mL) was treated with acetic anhydride (0.5 mL) and stirred for 24 h at room temperature. Extraction with EtOAc in the usual way and column chromatography afforded 6-acetoxy-tremetone (**1a**) (12.3 mg):

Amorphous solid; ^1^H NMR (500 MHz): δ 1.77 (3H, br s, H-12), 2.34 (3H, s, -OAc), 2.49 (3H, s, H-14), 3.05 (1H, dd, J= 15.7, 9.0 Hz, H-3α), 3.36 (1H, dd, J= 15.8, 9.0 Hz, H-3β), 4.95 and 5.09 (1H each, s, H-11), 5.29 (1H, t, J = 9.0, Hz, H-2β), 6.52 (1H, s, H-7), 7.68 (1H, s, H-4); ^13^C NMR (125 MHz): See Table 1; EIMS *m/z* (rel. int.): 260 [M]^+^ (17), 218 (78), 203 (100), 175 (57), 160 (11), 129 (7); HREIMS *m/z*: 260.1053 [M]^+^. Calculated for C_15_H_16_O_4,_ 260.1049.

### 3.8. Acetylation of Euparone (7)

Compound **7** (8 mg) in pyridine (0.3 mL) at room temperature was treated with Ac_2_O (0.3 mL) and stirred for 24 h. Usual work-up and column chromatography yielded 6-acetoxy-euparone (**7a**) (9.3 mg):

Colourless oil; ^1^H NMR (500 MHz): δ 2.39 (3H, s, -OAc), 2.62 (6H, s, H-11 and H-13), 7.34 (1H, s, H-7), 7.54 (1H, br s, H-3), 8.20 (1H, s, H-4; ^13^C NMR (125 MHz): See Table 1; EIMS *m/z* (rel. int.): 260 [M]^+^ (17), 218 (100), 203 (90), 175 (57), 160 (11), 129 (7); HREIMS *m/z*: 260.0689 [M]^+^. Calculated for C_14_H_12_O_5_, 260.0685.

### 3.9. Biotransformation of 6-Hydroxytremetone (1)

#### 3.9.1. Microorganism

The fungal strain *Mucor plumbeus* (CECT 20422) used in this work was obtained from Colección Española de Cultivos Tipo (CECT), Valencia, Spain.

#### 3.9.2. Incubation of 1

Two millilitres of a conidial suspension in water containing approximately 9 × 10^6^ conidia/mL of the fungus *M. plumbeus* was added to each 52 conical flasks (250 mL), containing sterile medium (50 mL) [40]. The cultures were grown at 25 °C for 36 h on an orbital shaker. The substrate 1 (265 mg) in EtOH (5.3 mL) was distributed equally between the flasks, and the incubation was allowed to continue for a further 6 days. The mycelium was separated by vacuum filtration and the broth extracted three times with EtOAc. The solvent was evaporated to give a residue (1.30 g), which was chromatographed on a silica gel column using hexane-EtOAc and EtOAc-MeOH gradients. Final purification of fractions of interest were carried out by semipreparative HPLC using n-hexane-EtOAc gradient as the mobile phase and flow rate 3 mL/min. Biotransformation of **1** gave in polarity order, starting material (**1**) (25.5 mg, 1.96% of extract), 6-hydroxytremetone β-D-glucoside (**18**) (145.0 mg, 11,5% of extract), and 6,10,11-trihydroxytremetone (**19**) (2.0 mg, 0.15% of extract).

Compound **18** (15 mg) in pyridine (0.6 mL) at room temperature was treated with Ac_2_O (0.6 mL) and stirred for 24 h. Extraction with EtOAc in the usual way and column chromatography afforded 6-hydroxytremetone β-D-tetraacetylglucoside (**18a**) (17.3 mg).

#### 3.9.3. 6-Hydroxytremetone β-D-Glucoside (18)

M.p.: 85.0–90.1 °C (EtOAc-MeOH); ^1^H NMR (500 MHz, CDCl_3_): δ 1.72 (3H, s, H-12), 2.46 (3H, s, H-14), 2.94 and 3.22 (each 1H, dd, *J =*15.8, 8.0 Hz, H-3), 3.47 (1H, d, *J =*10.0 Hz, H-5′), 3.68 (3H, br s, H-2′, H-3′ y H-4′), 3.88 (2H, br s, H-6′), 4.85 (1H, d, *J =*6.6 Hz, H-1′), 4.90 and 5.04 (each 1H, s, H-11), 5.20 (1H, t, *J =*8.0 Hz, H-2), 6.60 (1H, s, H-7), 7.47 (1H, s, H-4); ^1^H NMR (500 MHz, CD_3_OD): δ 1.76 (3H, s, H-12), 2.65 (3H, s, H-14), 3.00 and 3.37 (each 1H, dd, *J =*15.6, 8.0 Hz, H-3), 3.44 (2H, m, H-3′, H-4′), 3.50 (1H, dd, *J =*5.4, 2.2 Hz, H-5′), 3.55 (1H, m, H-2′), 3.75 (1H, dd, *J =*12.1, 5.4 Hz, H-6′), 3.93 (1H, dd, *J =*12.1, 2.2 Hz, H-6′), 4.93 and 5.09 (each 1H, s, H-11), 5.05 (1H, d, *J =*7.3 Hz, H-1′), 5.30 (1H, t, *J =*8.0 Hz, H-2), 6.75 (1H, s, H-7), 7.63 (1H, s, H-4); ^13^C NMR (125 MHz): See Table 3; EIMS *m/z* (rel. int.): 380 [M]^+^ (5), 218 (100), 203 (93), 185 (13), 175 (49), 160 (21), 91 (10); HR-EISI-MS *m/z*: 380.1471 [M]^+^. Calculated for C_19_H_24_O_8,_ 380.1471. *6-Hydroxytremetone β-D-tetraacetylglucoside* (**18a**): ^1^H NMR (500 MHz): δ 1.73 (3H, s, H-12), 2.02, 2.03, 2.05 and 2.09 (each 3H, s, -OAc), 2.48 (3H, s, H-14), 2.98 and 3.29 (each 1H, ddd, *J* =15.4, 8.0, 1.3 Hz, H-3), 3.88 (1H, ddd, *J* =10.0, 6.3, 2.5 Hz, H-5′), 4.17 (1H, dd, *J* =12.3, 2.5 Hz, H-6′), 4.26 (1H, dd, *J* =12.3, 6.3 Hz, H-6′), 4.92 and 5.05 (each 1H, br s, H-11), 5.16 (1H, t, *J* =11.0 Hz, H-4′), 5.18 (1H, d, *J* =9.5 Hz, H-1′), 5.24 (1H, br t, *J* =8.0 Hz, H-2), 5.30 (1H, t, *J* =9.0 Hz, H-3′), 5.34 (1H, m, H-2′), 6.51 (1H, s, H-7), 7.60 (1H, t, J =*1.3* Hz, H-4); ^13^C NMR (125 MHz): See Table 3; EIMS *m/z* (rel. int.): 548 [M]^+^ (1), 331 (25), 218 (10), 203 (13), 169 (100), 145 (8), 127 (23), 109 (65), 91 (6); HR-EISI-MS *m/z*: 548.1874 [M]^+^. Calculated for C_27_H_32_O_12_, 548.1894.

#### 3.9.4. 6,10,11-. Trihydroxytremetone (19)

Colourless oil; [α]_D_ = +8 (c, 0.13, CHCl_3_); ^1^H NMR (500 MHz): δ 1.19 (3H, s, H-12), 2.54 (3H, s, H-14), 3.19 (2H, dd, *J =*12.1, 7.6 Hz, H-3), 3.55 and 3.77 (each 1H, dd, *J =*10.6, 5.0 Hz, H-11), 4.90 (1H, dd, *J =*9.5, 7.6 Hz, H-2), 6.33 (1H, s, H-7), 7.51 (1H, s, H-4), 12.96 (1H, s, OH); ^13^C RMN (125 MHz): See Table 3; MS *m/z* (rel. int.): 252 [M]^+^ (71), 221 (12), 203 (56), 178 (100), 165 (21), 163 (48), 160 (24), 134 (68), 107 (19), 75 (35), 57 (24); HR-EISI-MS *m/z*: 252.1008 [M]^+^. Calculated for C_13_H_16_O_5_, 252.0998.

### 3.10. Insect Bioassays

#### 3.10.1. Choice Feeding Assays (<6 h)

*Spodoptera littoralis* colonies are maintained at ICA-CSIC, reared on artificial diet, and kept at 22 ± 1 °C and >70% RH, with a photoperiod of 16:8 h (L:D) in a growth chamber. The bioassays were conducted as described [52]. The upper surface of *Capsicum anuum* (1.0 cm^2^) were treated with 10 μL of the test substance. The products were tested at an initial dose of 5 µg/µL (50 µg/cm^2^). Five Petri dishes with two sixth-instar *S. littoralis* larvae (>24 h after moulting) were allowed to feed in a growth chamber (until 75% larval consumption of control disks or tretament disks, environmental conditions as above). Each experiment was repeated 2 times. Feeding inhibition was calculated by measuring the disk surface consumption (digitalized with https://imagej.nih.gov/ij/, accessed on 23 March 2020) as % FI = [1 − (T/C) × 100], where T and C represent feeding on treated and control leaf disks, respectively. The antifeedant effects (% FI) were analyzed for significance by the nonparametric Wilcoxon signed-rank test. Extracts and compounds with an FI > 70% were further tested in a dose-response experiment (4–5 serial dilutions) to calculate their relative potency (EC_50_, the effective dose to give a 50% settling reduction) from linear regression analysis (% FI on Log-dose).

#### 3.10.2. Cannulation

This experiment was performed with preweighed newly moulted *S. littoralis* L6 larvae in the absence or presence of piperonyl butoxide (PBO), under the same environmental conditions as above. Each experiment consisted of 20 larvae orally dosed with 40 and 20 µg of the test compound in 4 µL of DMSO (treatment) or solvent alone (control) using a Rheodyne Hamilton syringe (50 μL) attached to a Hamilton microdispenser. The syringe tip was inserted into the mouth of the larvae (maximum of 5 mm), and then larvae were forced fed until no regurgitation was observed. PBO-pretreated larvae were orally injected with 10 µg of PBO in 2 µL of DMSO, 1 h prior to the oral applicaton of the test [53]. At the end of the experiments (72 h), larval consumption and growth were calculated on a dry weight basis. The possible effect of variations in initial larval weight was analyzed by an analysis of covariance (ANCOVA) performed on biomass gains with initial biomass as covariate. The covariate effect was not significant (*p* > 0.05), showing that changes in insect biomass were similar among all treatments. An additional ANOVA was performed with ΔI as covariate for the extracts that signifcantly reduced ΔB, (ANCOVA2) to understand their post-ingestive mode of action (antifeedant and/or toxic) [54,55].

## 4. Conclusions

In this work, we have studied the benzofurans of *Pericallis echinata* (aerial parts and transformed roots), *P. steetzii* (aerial parts and transformed roots), *P. lanata* (aerial parts), and *P. murrayi* (aerial parts and roots). This work has permitted the isolation of the new compounds 10-ethoxy-11-hydroxy-10,11-dihydroeuparin (**10**), (-)-eupachinin A ethyl ether (**12**), 11,15-didehydro-eupachinin A (**13**), 10,12-dihydroxy-11-angelyloxy-10,11-dihydroeuparin (**14**), 11-angelyloxy-10,11-dihydroeuparin (**16**), 12-angelyloxyeuparone (**17**) and 2,4-dihydroxy-5-formyl-acetophenone (**15**) isolated for the first time as a natural product along with several known ones (**1**–**9, 11**). The incubation of the abundant component, 6-hydroxytremetone (**1**), with the fungus *Mucor plumbeus* afforded two benzofuran derivatives, the new compound 6-hydroxytremetone β-D-glucoside (**18**), and 6,10,11-trihydroxytremetone (**19**).

Benzofurans in the tremetone series (**1**, **1a**, **2**–**5**, **18**, **18a**), the euparin series (**6**, **7**, **7a**, **8**–**10**, **14**, **16**), and the eupachinin-type (**11**, **12**) were tested for antifeedant effects against the insect *Spodoptera littoralis*. In this work, ethoxylation of the C3 hydroxy group in the furan ring gave the strongest antifeedant effect (**4**), while acetylation of the aromatic hydroxyl group reduced or eliminated the antifeedant activity in the tremetone series (**1** vs. **1a**). Modifications of the side chain also affected the activity. For example, the oxydation of euparin **6** in C10 eliminated the activity (in **7** and **7a**) and the presence of a C11 angelate substituent in the side chain (**16**, **14**) increased the activity (**8**)**.**

Compounds **4, 11,** and **12** showed antifeedant effects without postingestive toxicity to orally dosed *S. littoralis* larvae. In this work, we have shown growth inhibitory effects on *S. littoralis* larvae for the tremetone derivative **4** and eupachinin compounds **11** and **12,** while euparin **6**, with an unsaturated benzofuran ring, was the most toxic compound. The larval toxicity of **6** was enhanced by the application of the synergist PBO, indicating that insects can detoxify these compounds.

## Figures and Tables

**Table 1:** ^13^C NMR data of compounds **1**–**7**, **1a**, and **7a**.

Carbon	1	1a	2	3	4	5	6	7	7a
2	87.6	87.8	95.8	92.1	92.8	90.6	157.9	153.7	154.6
3	33.1	33.7	75.5	83.1	81.9	71.0	102.4	113.1	112.6
4	126.6	127.0	128.7	129.1	129.3	129.1	123.5	126.9	127.6
5	113.8	123.0	114.6	114.4	114.4	114.8	116.9	118.4	128.3
6	165.8	164.0	167.1	167.0	167.1	166.9	161.6	163.4	149.6
7	98.1	105.1	98.7	98.5	98.5	99.0	99.2	100.4	108.1
8	166.6	151.3	166.6	167.0	167.0	166.1	159.7	160.0	157.0
9	118.6	124.8	120.3	117.1	118.4	120.9	121.9	119.7	124.7
10	143.2	142.9	140.8	141.1	141.3	138.7	132.1	188.0	188.1
11	112.7	112.9	113.2	113.1	113.2	113.7	113.7	26.4	26.5
12	17.0	17.1	17.4	17.4	17.5	20.0	19.2	204.0	196.7
13	201.9	195.6	202.3	202.2	202.3	202.5	203.9	26.9	26.5
14	26.2	29.2	26.3	26.1	26.2	26.3	26.8		
1′	-		-	55.3	63.6	-	-	-	
2′	-		-	-	15.4	-	-	-	
AcO-C(6)		169.5 21.2							169.4 21.1

**Table 2:** ^13^C NMR data of compounds **8**–**12**, **14,** and **16**.

Carbon	8	9	10	11	12	14	16
2	159.5	159.6	159.1	171.0	170.8	157.6	160.8
3	102.8	105.3	104.7	115.8	116.1	104.6	101.8
4	123.6.	123.7	123.6	125.2	125.3	123.8	122.9
5	117.0.	117.1	117.0	118.2	118.2	117.2	116.6
6	161.1	165.0	161.2	162.0	162.0	161.2	160.8
7	99.8	99.9	99.8	100.4	100.4	99.9	99.5
8	161.1	161.3	159.5	159.0	159.0	159.5	159.4
9	121.0	120.7	120.8	115.1	115.9	120.6	121.3
10	72.2	73.0	76.2	194.1	194.8	74.1	33.3
11	68.8	68.2	68.5	35.7	35.5	66.2	66.2
12	23.4	18.0	18.7	38.2	36.8	65.3	15.5
13	204.0	204.0	203.9	67.4	71.3	204.0	203.9
14	26.8	26.8	26.8	25.8	21.8	26.8	26.8
15	-	-	-	204.6	204.6	-	-
16	-	-	-	27.2	27.2	-	-
1′	-	51.1	58.9	-	59.9	168.3	167.7
2′	-	-	15.7	-	15.8	126.9	127.5
3′	-	-	-	-	-	140.1	138.4
4′	-	-	-	-	-	20.4	20.5
5′	-	-	-	-	-	15.8	15.7

**Table 3:** ^13^C NMR data of compounds **18**, **18a** and **19**.

Carbon	18 ^b^	18 ^c^	18a ^d^	19
**2**	87.7	88.9	87.8	86.7
**3**	33.4	34.3	33.4	28.7
**4**	126.9	127.6	126.8	126.7
**5**	121.3	122.6	122.4	114.0
**6**	158.9	160.8	157.5	165.2
**7**	98.7	98.3	96.6	98.2
**8**	164.6	166.5	164.5	166.2
**9**	121.5	122.6	121.8	118.7
**10**	143.1	145.2	143.1	73.5
**11**	112.8	112.6	112.6	67.0
**12**	17.1	17.2	17.0	19.0
**13**	199.0	200.7	197.8	202.1
**14**	30.6	32.1	31.6	26.2
**1′**	102.5	102.6	98.6	
**2′**	73.3	74.8	71.0	
**3′**	76.2	78.3	72.9	
**4′**	69.5	71.2	68.2	
**5′**	76.0	78.2	72.3	
**6′**	61.7	62.4	61.9	

^b^ CDCl_3_, ^c^ CD_3_OD. ^d^ Acetates (**18a**): δ 169.2, 169.3, 170.1, 170.5 (CO); 20.5 (Me).

**Table 4:** Insect antifeedant and postingestive effects of compounds **1–8**, **10–12**, **14**, **16**, **18,** and **18a**.

Compound	*Spodoptera littoralis* L6 Larvae					
	Antifeedant		Postingestive			
	%FI ^a^	EC_50_ (µg/μL) ^b^	µg/larvae	ΔB ^c^	ΔI ^d^	pANCOVA2
**1**	83.8 ± 3.5 ^e^	0.50 (0.24–1.03)		91.41 ± 1.17	88.39 ± 1.16	
**1a**	72.5 ± 9.16 ^e^	nc				
**2**	37.55 ± 3.11					
**3**	50.3 ± 7.5					
**4**	90.4 ± 6.2 ^e^	0.13 (0.05–0.34)	40	39.95 ± 15.03 ^g^	55.21 ± 10.94 ^g^	0.051
			20	106.39 ± 0.77	92.63 ± 0.76	
**6**	74.2 ± 7.8 ^e^	19.7 (11.5–34.0)	40	66.16 ± 1.11 ^g^	96.12 ± 1.56	0.035
			20	80.85 ± 0.87 ^g^	102.38 ± 0.94	0.465
			20 + 10 PBO ^h^	59.29 ± 1.17 ^g^	83.76 ± 1.51 ^g^	0.298
**7**	5.37 ± 4.47					
**7a**	1.00 ± 1.00					
**8**	8.15± 8.15					
**10**	29.50 ± 7.70					
**11**	98.5 ± 0.9 ^e^	1.04 (0.49–2.20)	40 ^f^	51.56 ± 16.76 ^g^	68.63 ± 12.99 ^g^	0.082
**12**	89.8± 6.5 ^e^	2.94 (2.21–3.91)	40 ^f^	43.00 ± 18.78 ^g^	69.47 ± 13.15 ^g^	0.135
**14**	83.6 ± 6.6 ^e^	0.84 (0.33–0.47)				
**16**	64.13 ± 8.29					
**18**	42.7 ± 13.2					
**18a**	8.06 ± 3.50					

^a^ Percent feeding inhibition (%FI, 5 µg/L); ^b^ EC_50_, efficient dose to give 50% activity (µg/L) and 95% confidence limits (lower, upper); ^c^ ΔB, change in larval body mass expressed as percent of the control; ^d^ ΔI; change in larval ingestion expressed as percent of the control; ^e^ *p* < 0.05, Wilcoxon Signed Rank Test; ^f^ N = 10 larvae; ^g^ pANCOVA1 < 0.05; ^h^ PBO (piperonyl butoxide).

## Data Availability

The data presented in this study are available in the Research Group databases, within the article and its Supplementary Material.

## Supplementary Materials

The following are available online at https://www.mdpi.com/article/10.3390/molecules28030975/s1, Figures S1–S2: ^1^H-NMR and ^13^C-NMR of compound 1a; Figure S3: ^13^C-NMR of compound 2; Figures S4–S5: ^13^C-NMR of compounds 4–5; Figures S6–S7: ^1^H-NMR an ^13^C-NMR of compound 7a; Figures S8–S10: ^13^C-NMR of compounds 8–10; Figures S11–S20: ^1^H-NMR and ^13^C-NMR of compounds 11–14 and 15–16; Figure S21: ^1^H-NMR of compound 17; Figures S22–S27: ^1^H-NMR and ^13^C-NMR of compounds 18,18a and 19.

## Sample Availability

Samples of the compounds are not available from the authors.
