# Supplementary material for: Insect Antifeedant Benzofurans from Pericallis Species
Source: Molecules. 2023 Jan 18;28(3):975. doi: 10.3390/molecules28030975 (PMC9918998; doi:10.3390/molecules28030975)
Supplement: Supplementary file 1 [file molecules-28-00975-s001.zip › molecules-2084562-supplementary.pdf]

Insect antifeedant benzofurans from *Pericallis* species

Carmen E. Díaz[a]*, Braulio M. Fraga[a], Adriana G. Portero[a], Iván Brito[b], Carmen López-Balboa[d], Liliana Ruiz-Vásquez[c] and Azucena González-Coloma[d]*

[a] *Instituto de Productos Naturales and Agrobiología, Consejo Superior de Investigaciones Científicas (CSIC), Avda. Astrof. F. Sánchez, 3, 38206 La Laguna, Tenerife, Spain*

[b] *Departamento de Química, Universidad de Antofagasta, Antofagasta 124000, Chile*

[c] *Laboratorio de Productos Naturales Antiparasitarios de la Amazonia, Centro de Investigación de Recursos Naturales, Universidad Nacional de la Amazonia Peruana (UNAP), 160001 Iquitos, Peru*

[d] *Instituto de Ciencias Agrarias, Consejo Superior de Investigaciones Científicas (CSIC), Serrano 115 dpdo., 28016 Madrid, Spain*

---

* Corresponding authors.

e-mail address: celisa@ipna.csic.es (C.E. Diaz)

e-mail address: azu@ica.csic.es (A. Gonzalez-Coloma)

List of contents:

Figure S1. ^{1}H-NMR spectrum of compound **1a** ($CDCl_3$, 500 MHz)

Figure S2. ^{13}C-NMR spectrum of compound **1a** ($CDCl_3$, 125 MHz)

Figure S3. ^{13}C-NMR spectrum of compound **2** ($CDCl_3$, 125 MHz)

Figure S4. ^{13}C-NMR spectrum of compound **4** (CDCl3, 125 MHz)

Figure S5. ^{13}C-NMR spectrum of compound **5** (CDCl3, 125 MHz)

Figure S6. ^{1}H-NMR spectrum of compound **7a** ($CDCl_3$, 500 MHz)

Figure S7. ^{13}C-NMR spectrum of compound **7a** ($CDCl_3$, 125 MHz)

Figure S8. ^{13}C-NMR spectrum of compound **8** ($CDCl_3$, 125 MHz)

Figure S9. ^{13}C-NMR spectrum of compound **9** ($CDCl_3$, 125 MHz)

Figure S10. ^{13}C-NMR spectrum of compound **10** ($CDCl_3$, 125 MHz)

Figure S11. ^{1}H-NMR spectrum of compound **11** (CDCl3, 500 MHz)

Figure S12. ^{13}C-NMR spectrum of compound **11** ($CDCl_3$, 125 MHz)

Figure S13. ^{1}H-NMR spectrum of compound **12** ($CDCl_3$, 500 MHz)

Figure S14. ^{13}C-NMR spectrum of compound **12** (CDCl3, 125 MHz)

Figure S15. ^{1}H-NMR spectrum of compound **14** (CDCl3, 500 MHz)

Figure S16. ^{13}C-NMR spectrum of compound **14** ($CDCl_3$, 125 MHz)

Figure S17. ^{1}H-NMR spectrum of compound **15** (CDCl3, 500 MHz)

Figure S18. ^{13}C-NMR spectrum of compound **15** ($CDCl_3$, 125 MHz)

Figure S19. ^{1}H-NMR spectrum of compound **16** (CDCl3, 500 MHz)

Figure S20. ^{13}C-NMR spectrum of compound **16** ($CDCl_3$, 125 MHz)

Figure S21. ^{1}H-NMR spectrum of compound **17** (CDCl3, 200 MHz)

Figure S22. ^{1}H-NMR spectrum of compound **18** (CDCl3, 500 MHz)

Figure S23. ^{13}C-NMR spectrum of compound **18** ($CDCl_3$, 125 MHz)

Figure S24. ^{1}H-NMR spectrum of compound **18a** (CDCl3, 500 MHz)

Figure S25. ^{13}C-NMR spectrum of compound **18a** ($CDCl_3$, 125 MHz)

Figure S26. ^{1}H-NMR spectrum of compound **19** (CDCl3, 500 MHz)

Figure S27. ^{13}C-NMR spectrum of compound **19** ($CDCl_3$, 125 MHz)

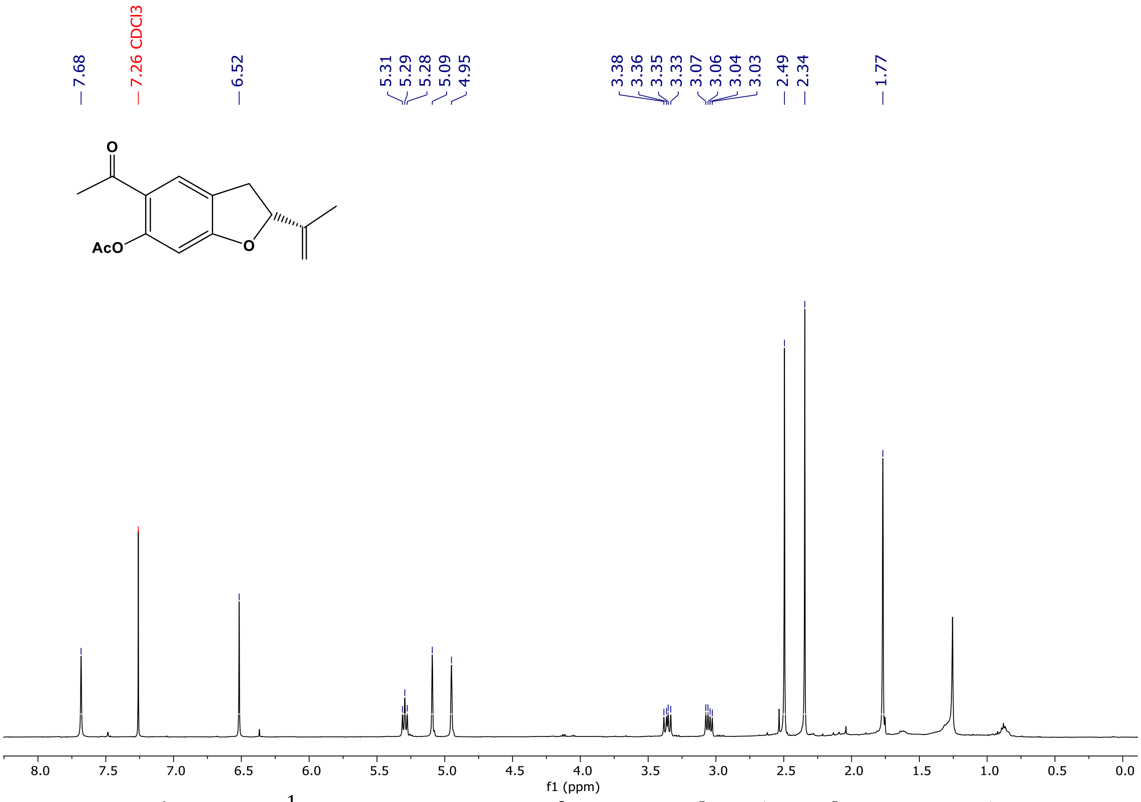

Figure S1. ^{1}H-NMR spectrum of compound **1a** ($CDCl_3$, 500 MHz)

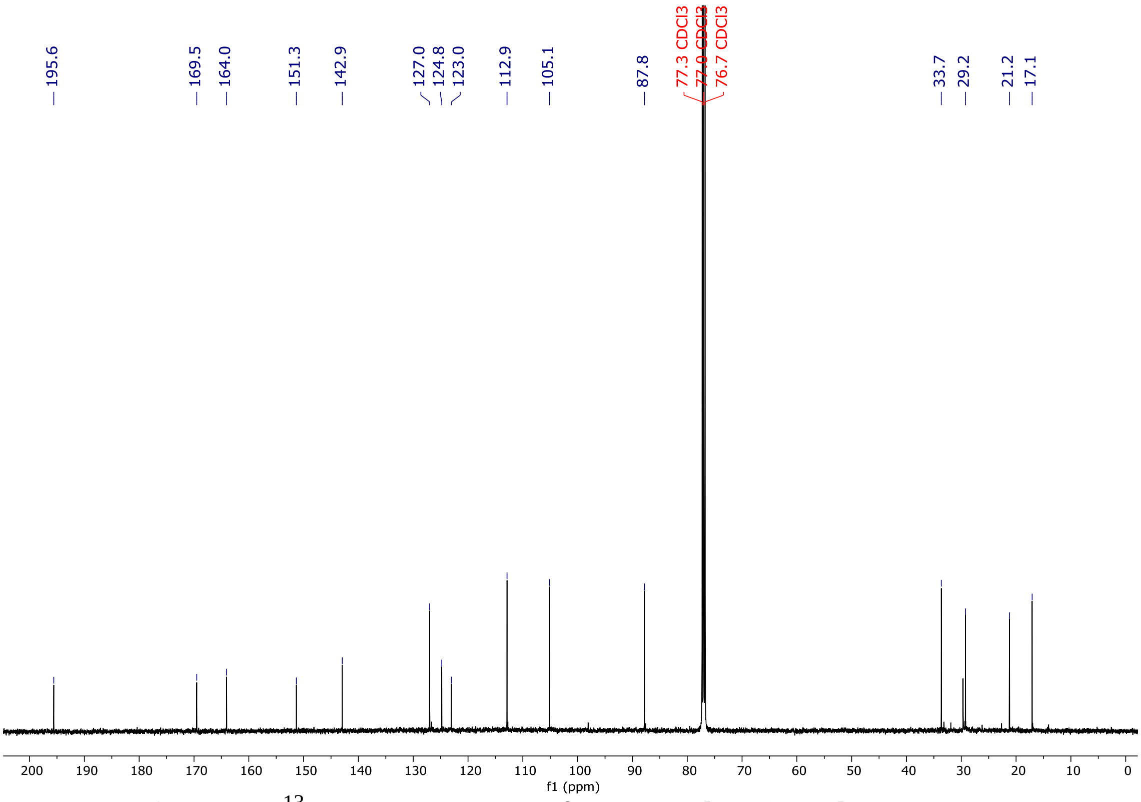

Figure S2. ^{13}C-NMR spectrum of compound **1a** ($CDCl_3$, 125 MHz)

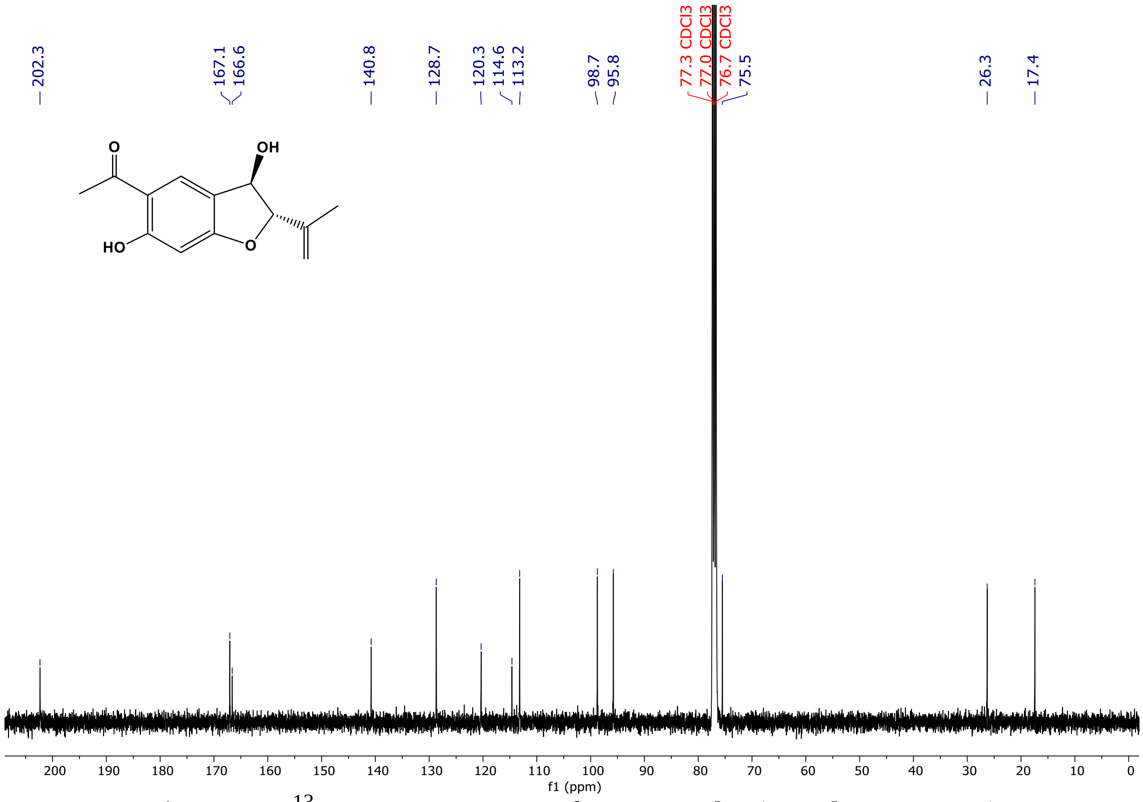

Figure S3. ^{13}C-NMR spectrum of compound **2** ($CDCl_3$, 125 MHz)

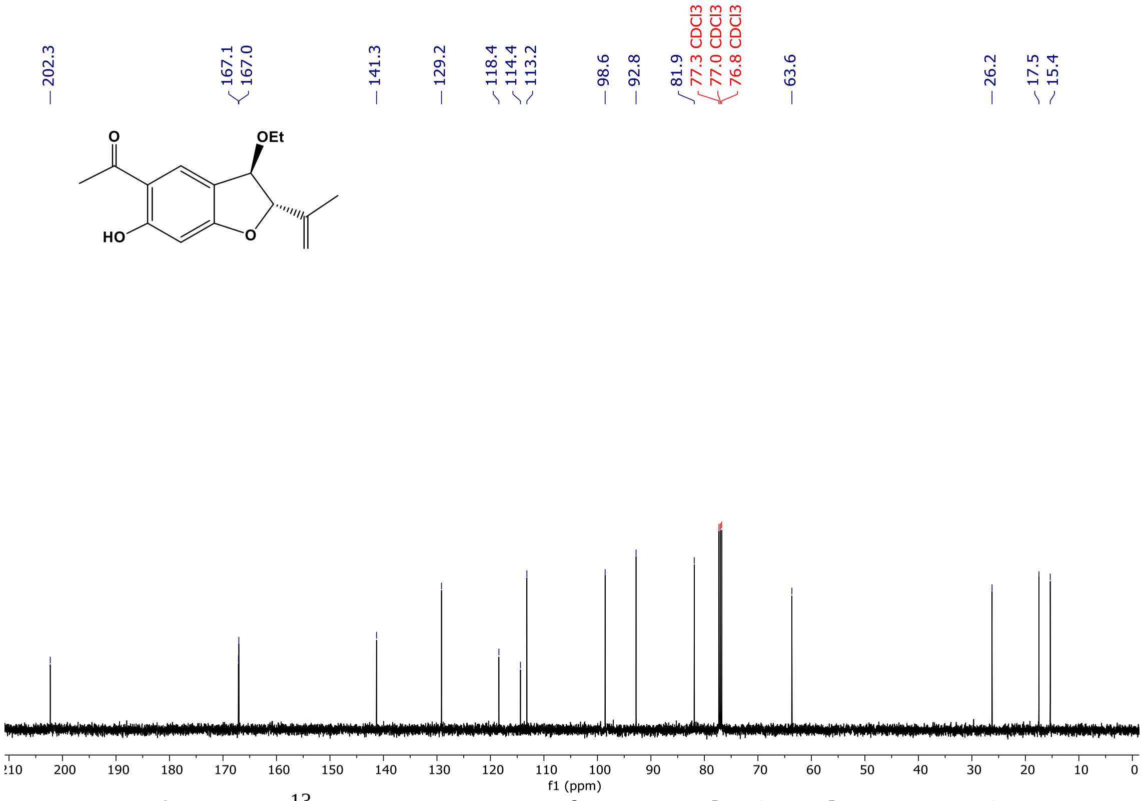

Figure S4. ^{13}C-NMR spectrum of compound **4** (CDCl3, 125 MHz)

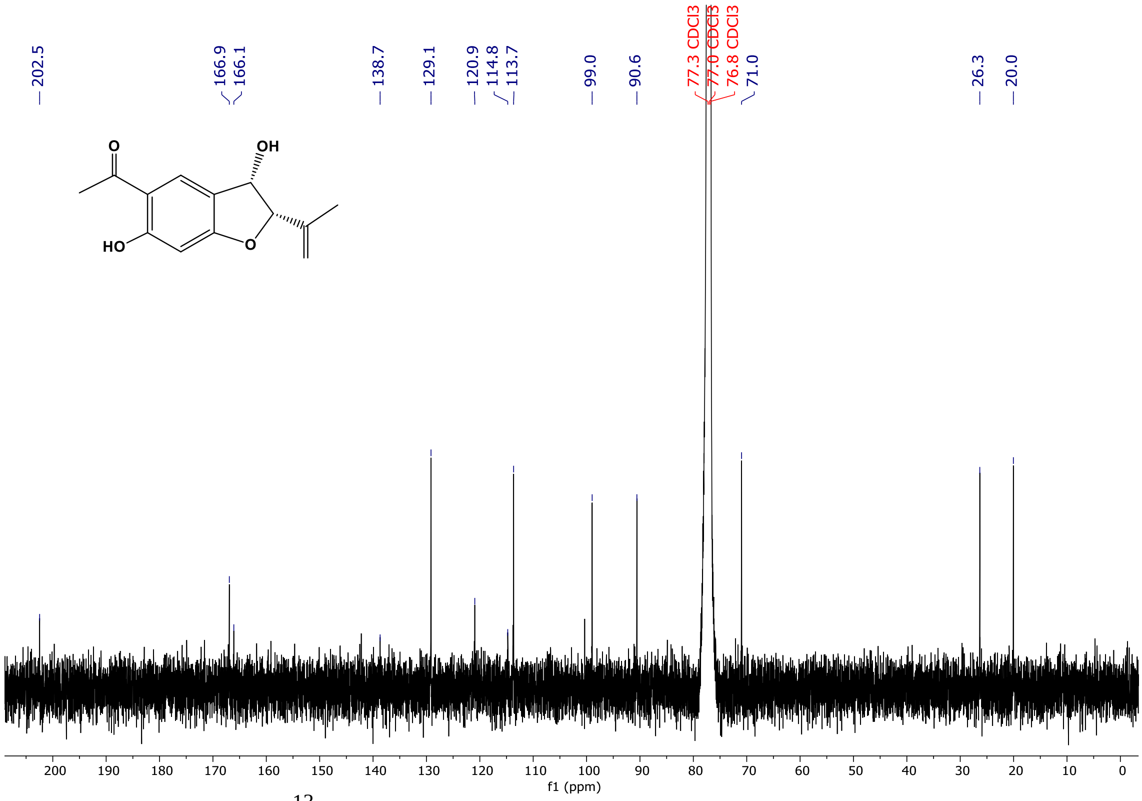

Figure S5. ^{13}C-NMR spectrum of compound **5** ($CDCl_3$, 125 MHz)

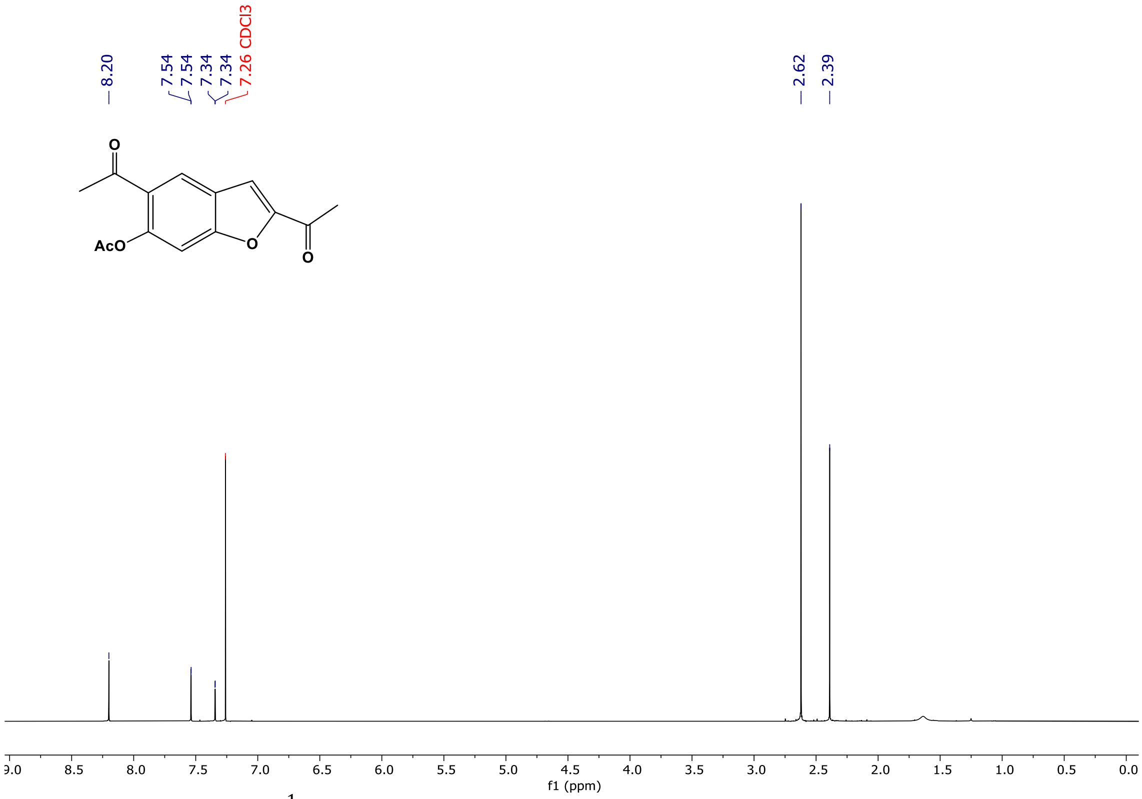

Figure S6. ^{1}H-NMR spectrum of compound **7a** (CDCl3, 500 MHz)

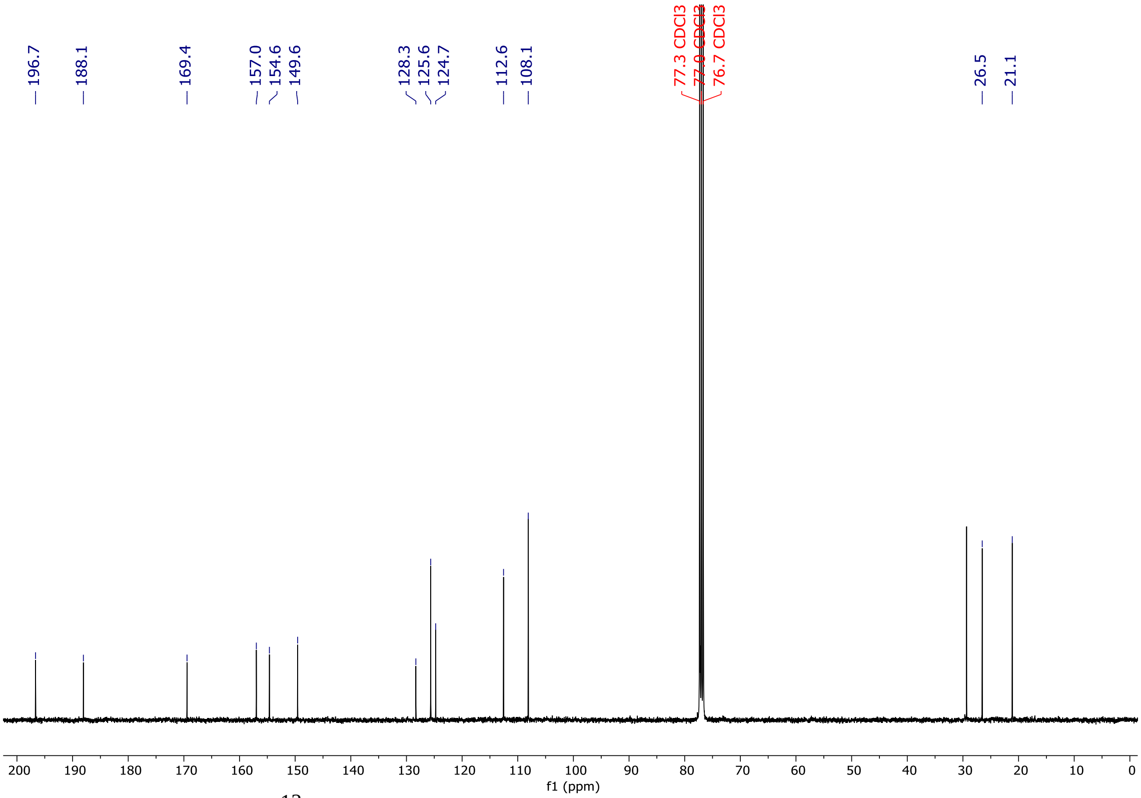

Figure S7. ^{13}C-NMR spectrum of compound **7a** (CDCl3, 125 MHz)

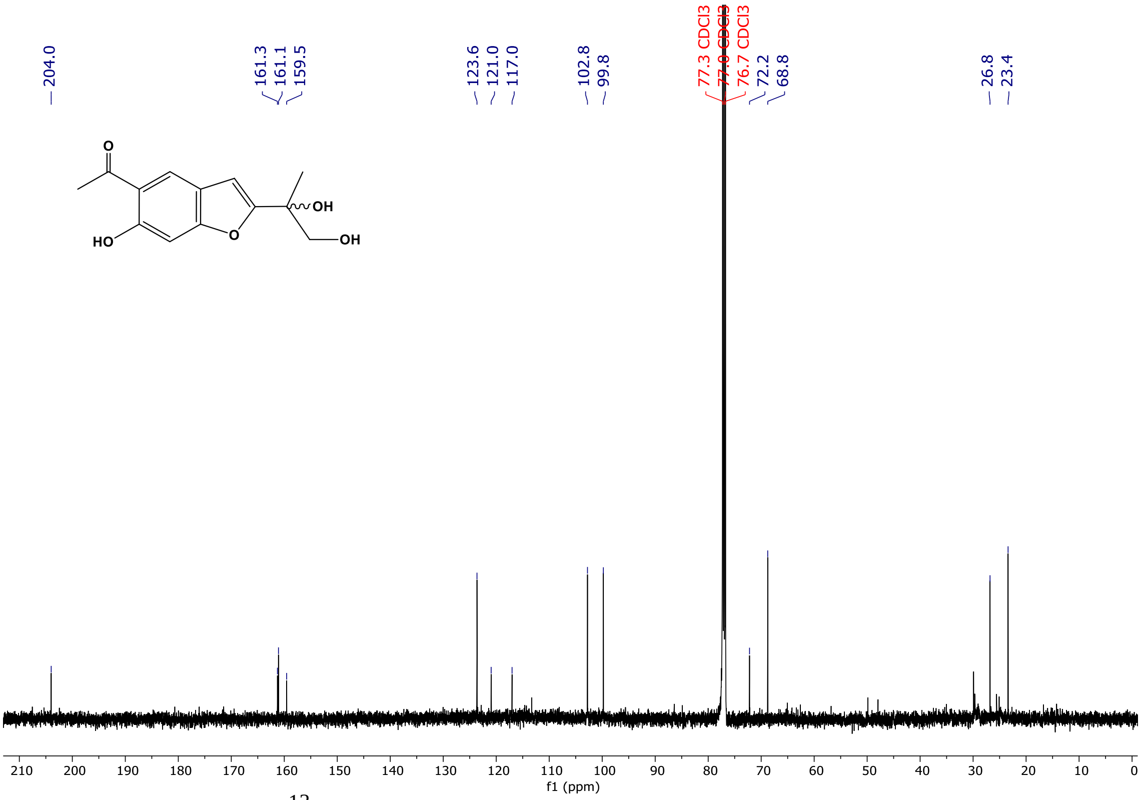

Figure S8. ^{13}C-NMR spectrum of compound **8** (CDCl3, 125 MHz)

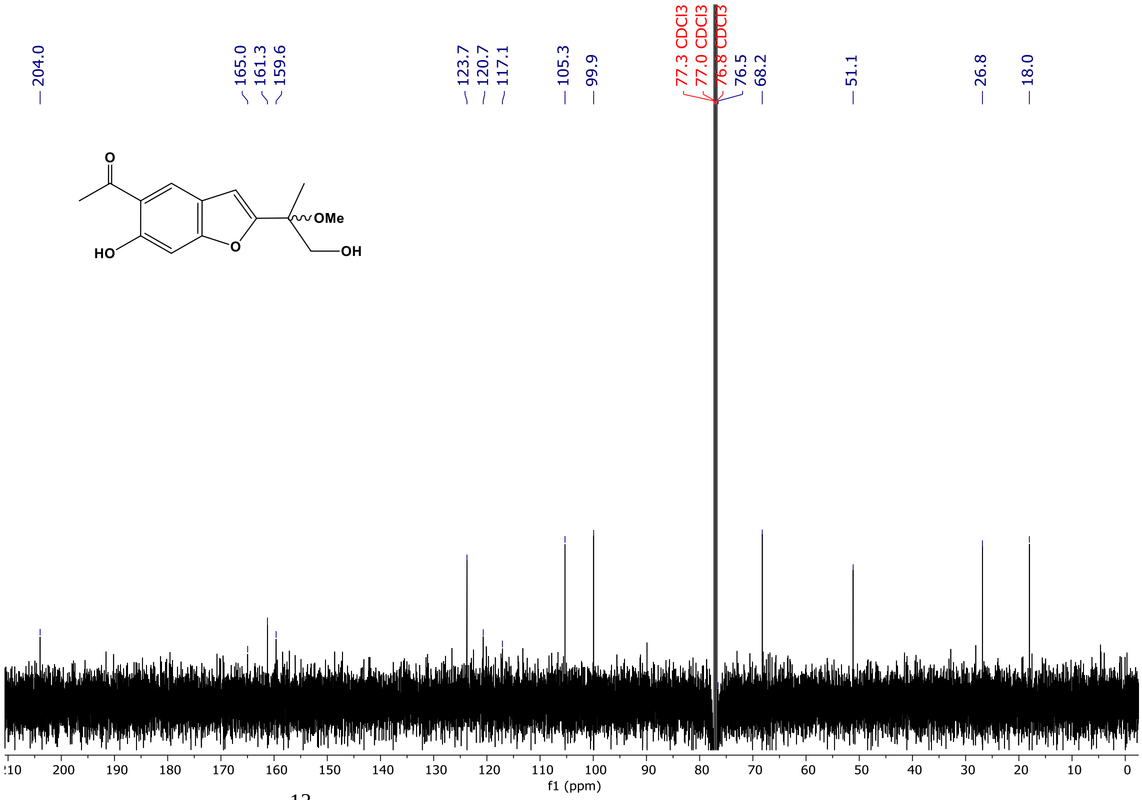

Figure S9. ^{13}C-NMR spectrum of compound **9** (CDCl3, 125 MHz)

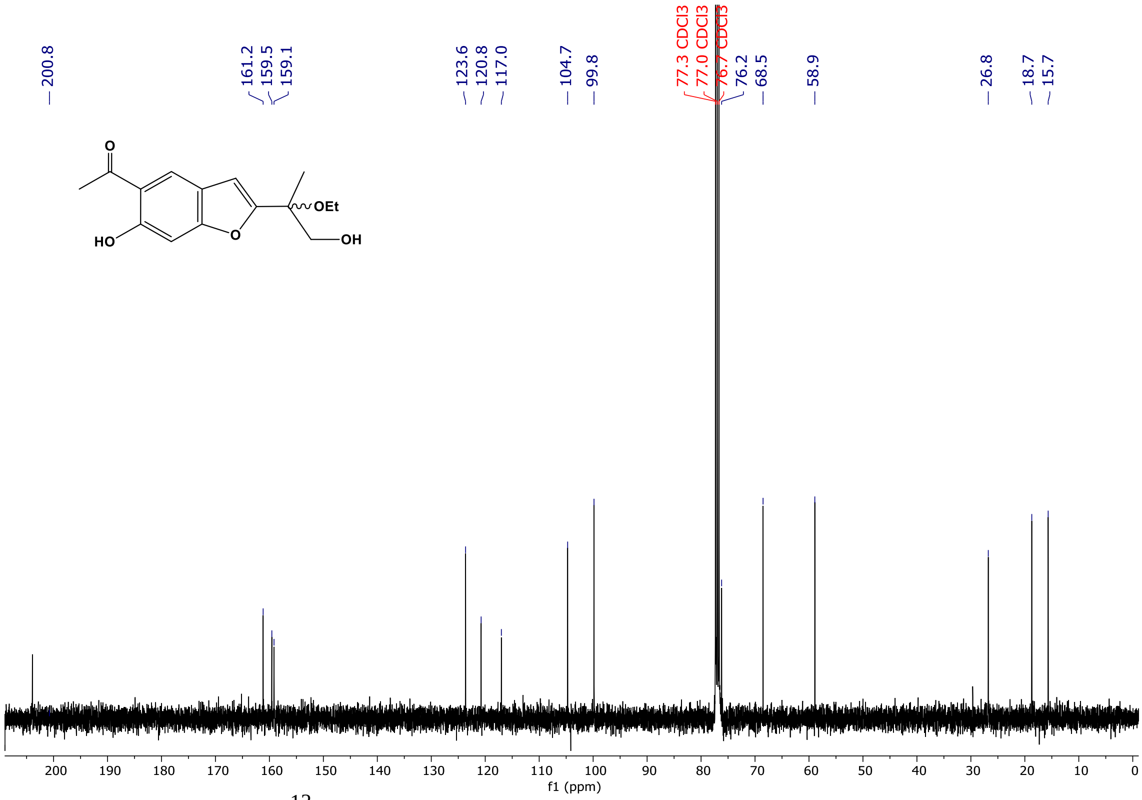

Figure S10. ^{13}C-NMR spectrum of compound **10** (CDCl3, 125 MHz)

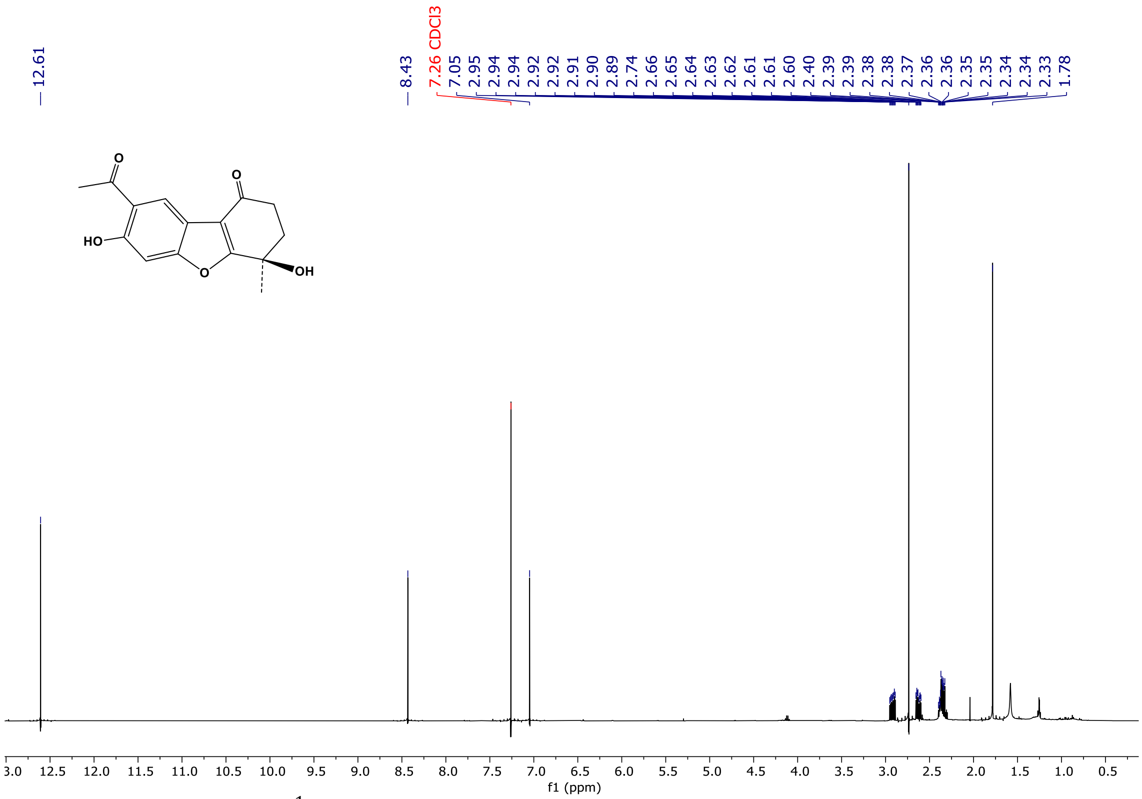

Figure S11. ^{1}H-NMR spectrum of compound **11** (CDCl3, 500 MHz)

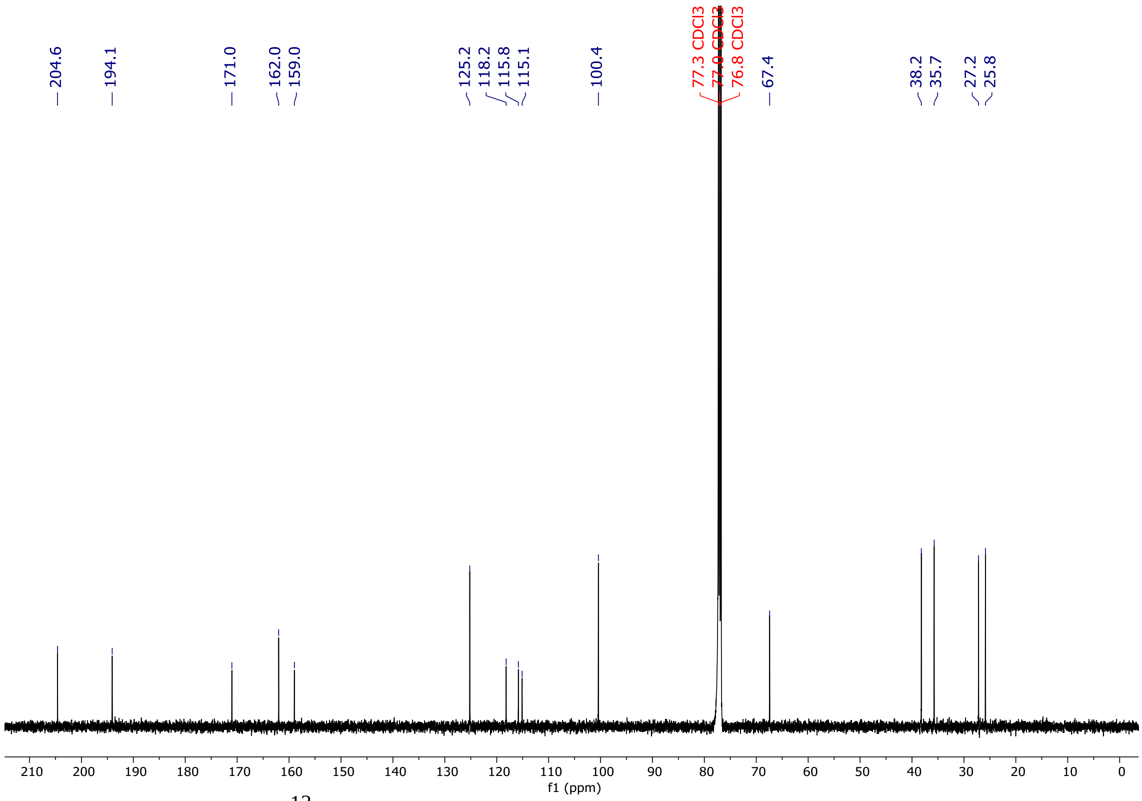

Figure S12. ^{13}C-NMR spectrum of compound **11** (CDCl3, 125 MHz)

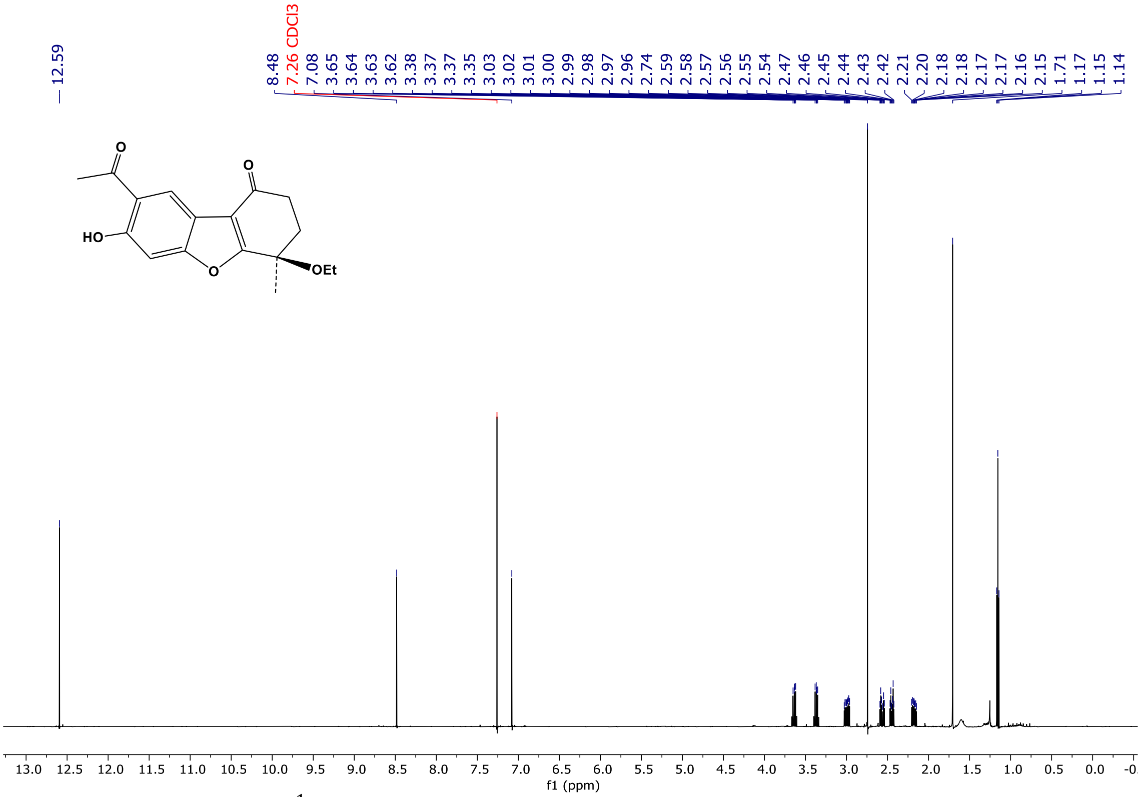

Figure S13. ^{1}H-NMR spectrum of compound **12** ($CDCl_3$, 500 MHz)

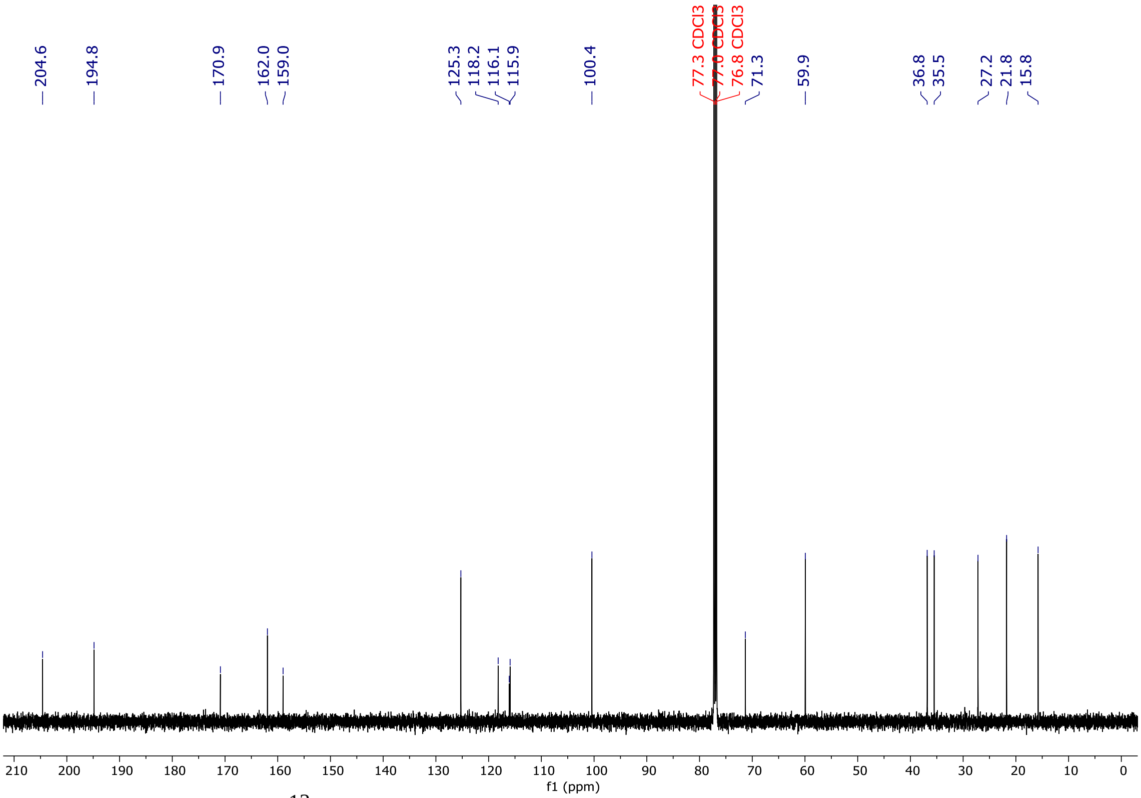

Figure S14. ^{13}C-NMR spectrum of compound **12** (CDCl3, 125 MHz)

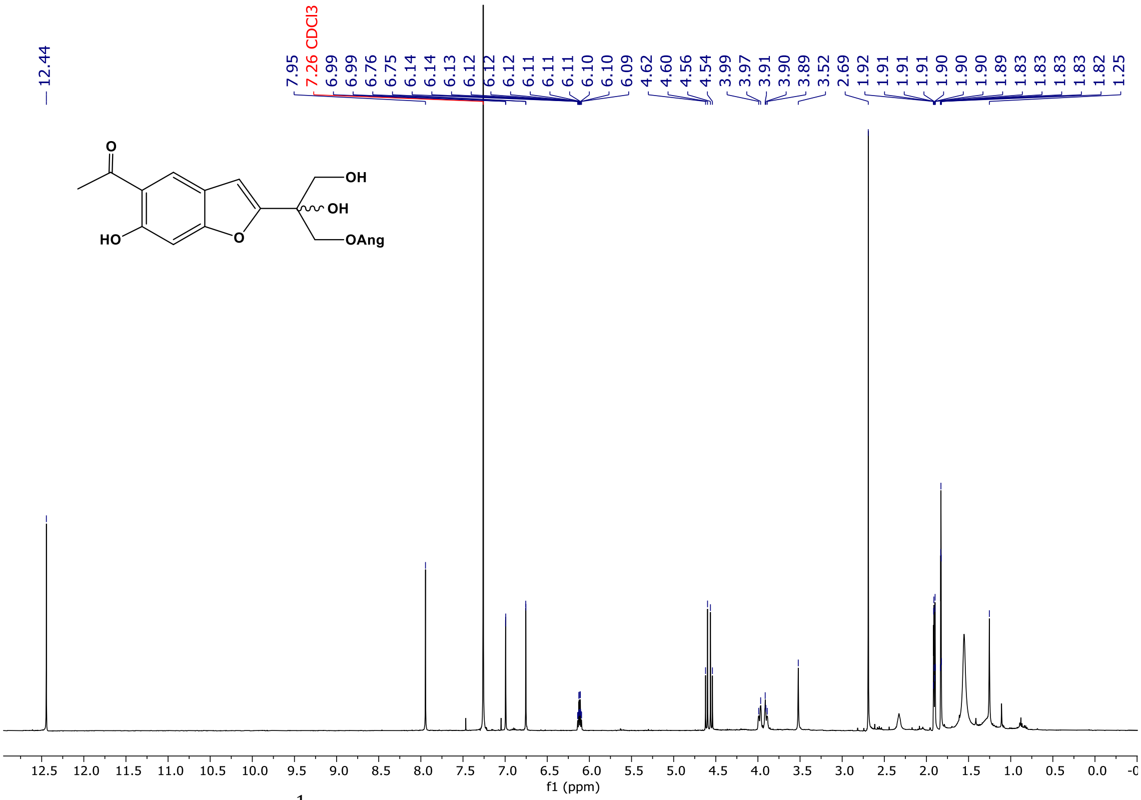

Figure S15. ^{1}H-NMR spectrum of compound **14** ($CDCl_3$, 500 MHz)

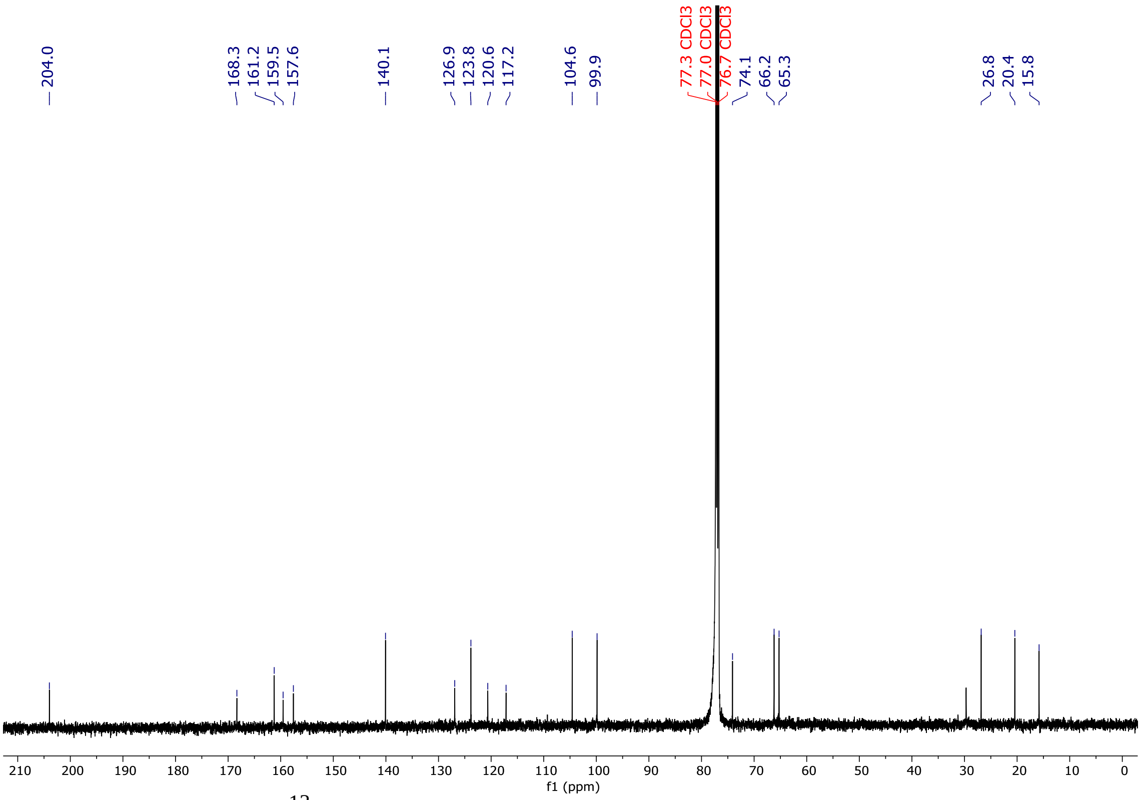

Figure S16. ^{13}C-NMR spectrum of compound **14** (CDCl3, 125 MHz)

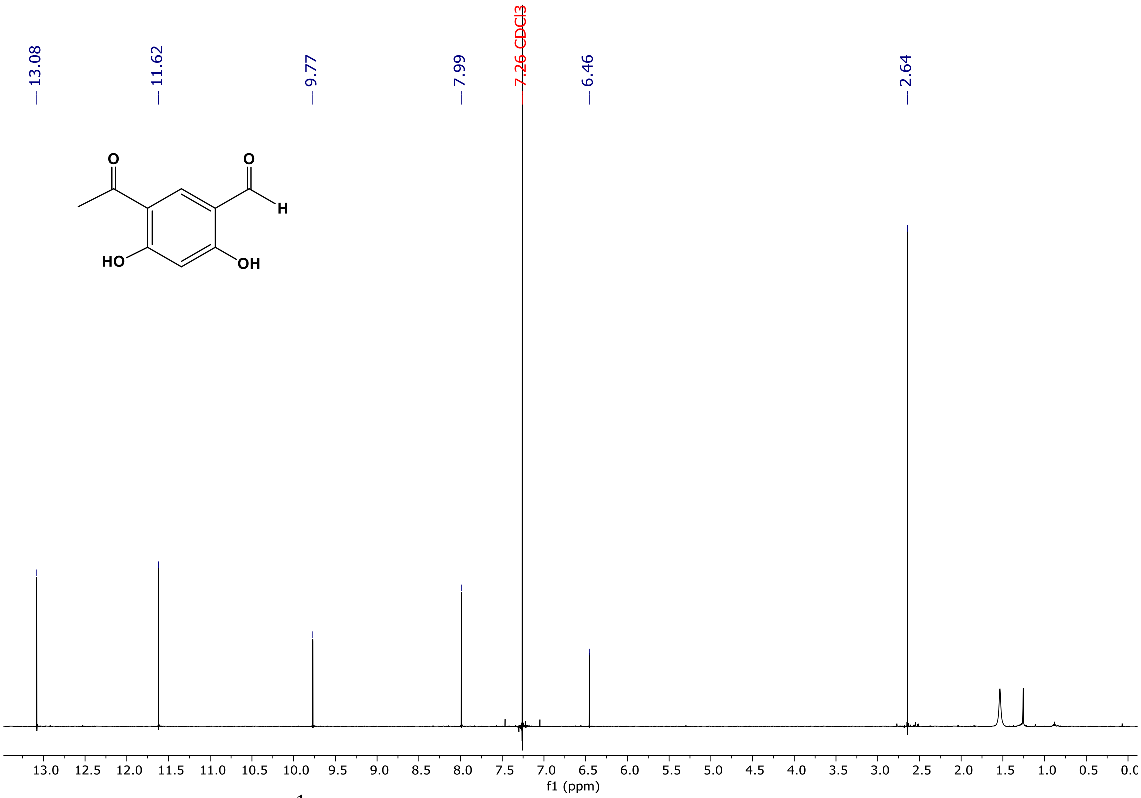

Figure S17. ^{1}H-NMR spectrum of compound **15** ($CDCl_3$, 500 MHz)

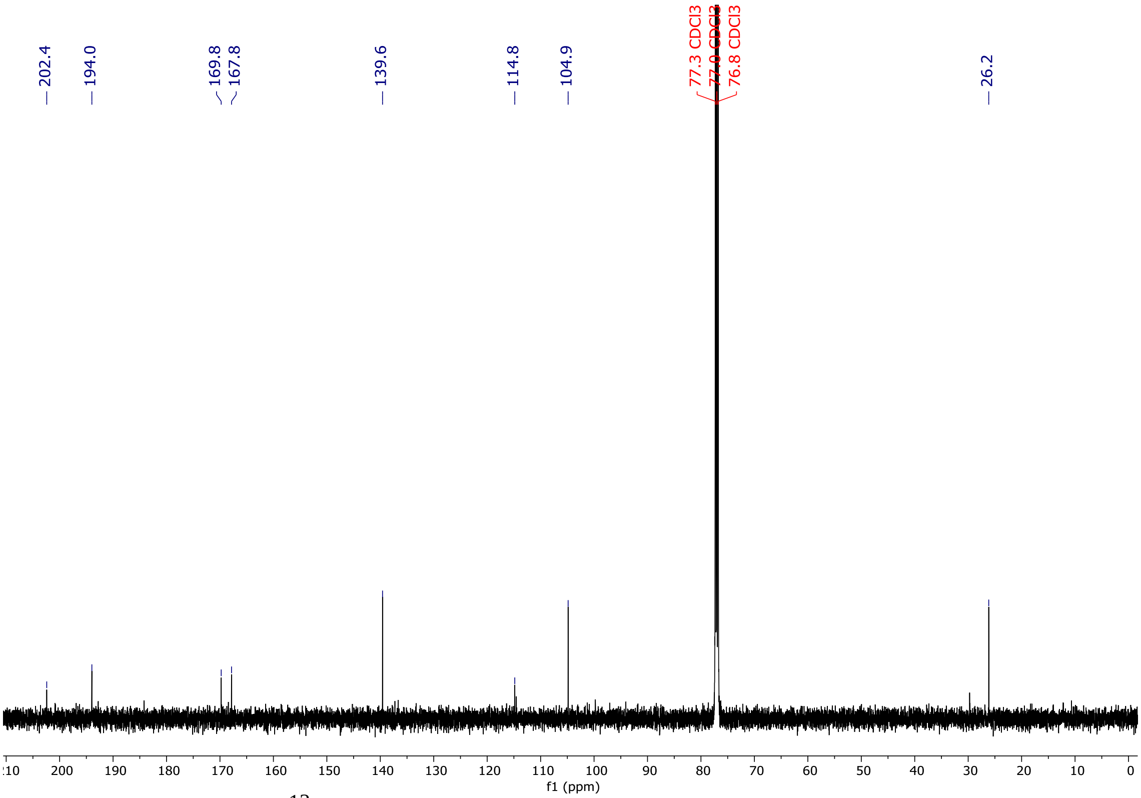

Figure S18. ^{13}C-NMR spectrum of compound **15** (CDCl3, 125 MHz)

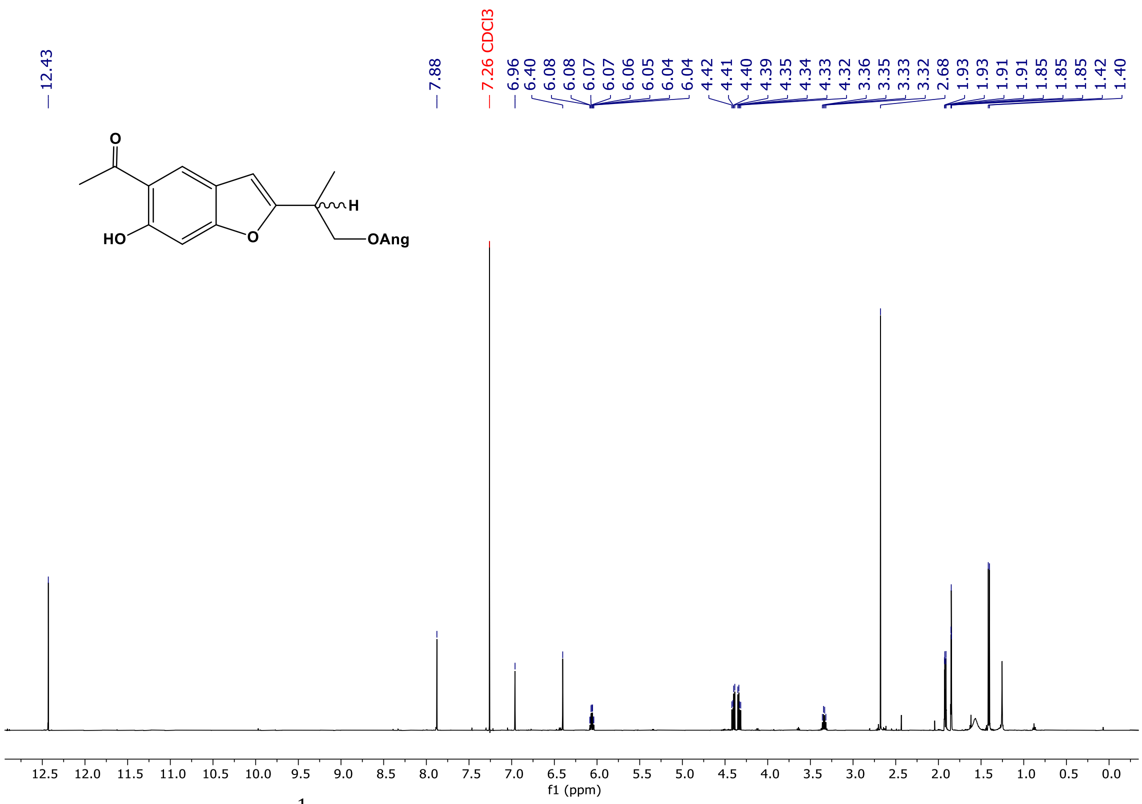

Figure S19. ^{1}H-NMR spectrum of compound **16** ($CDCl_3$, 500 MHz)

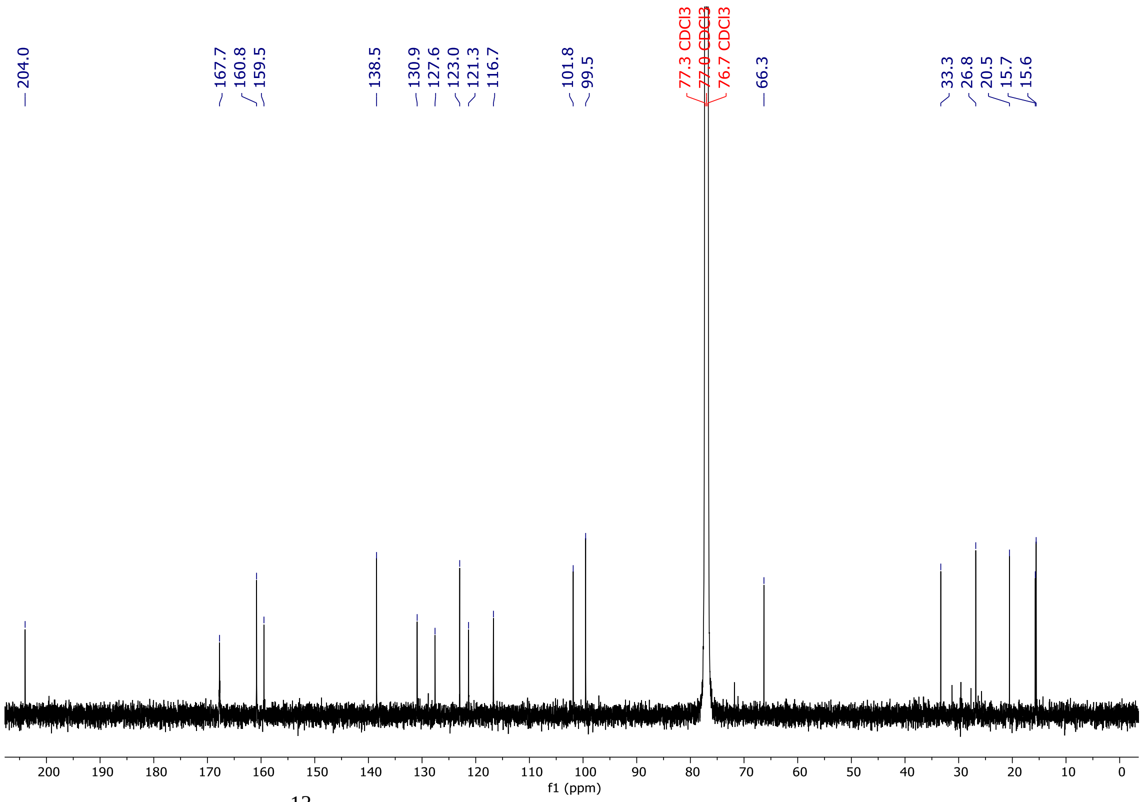

Figure S20. ^{13}C-NMR spectrum of compound **16** (CDCl3, 125 MHz)

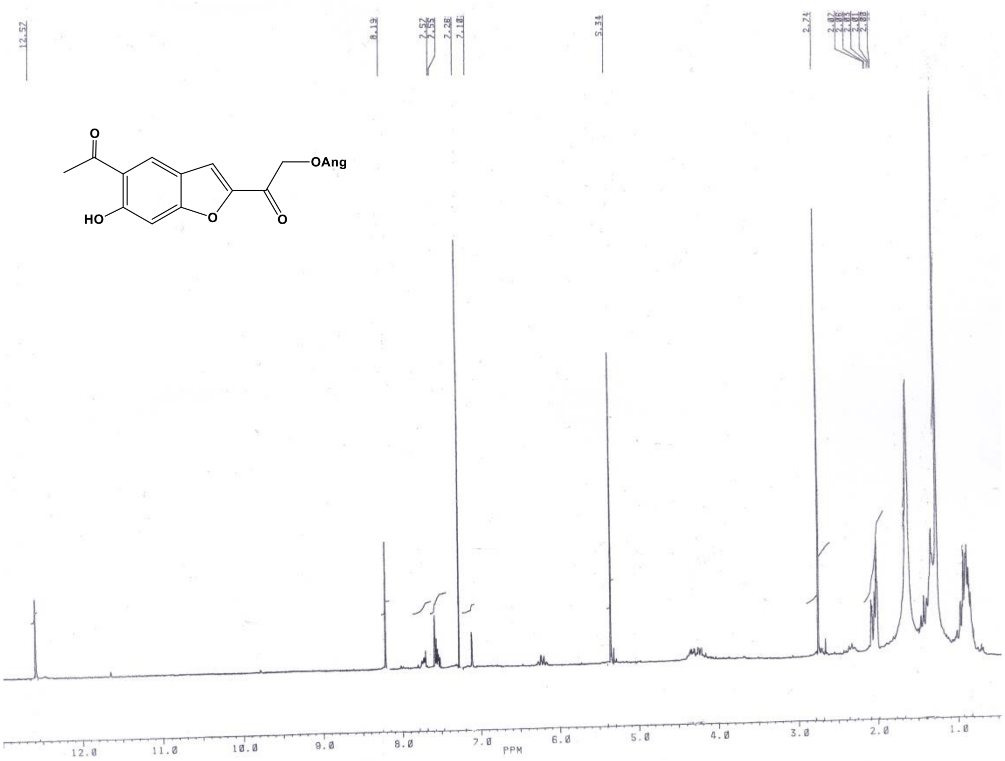

Figure S21. ^{1}H-NMR spectrum of compound **17** ($CDCl_3$, 200 MHz)

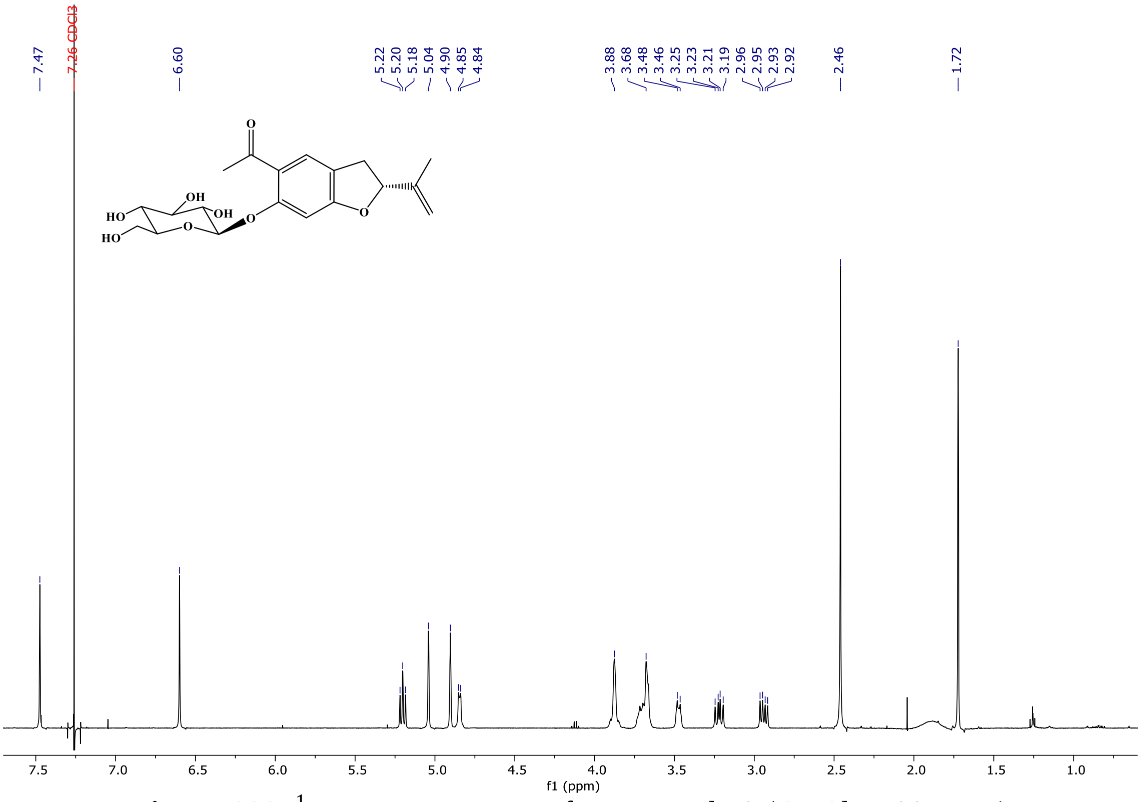

Figure S22. ^{1}H-NMR spectrum of compound **18** ($CDCl_3$, 500 MHz)

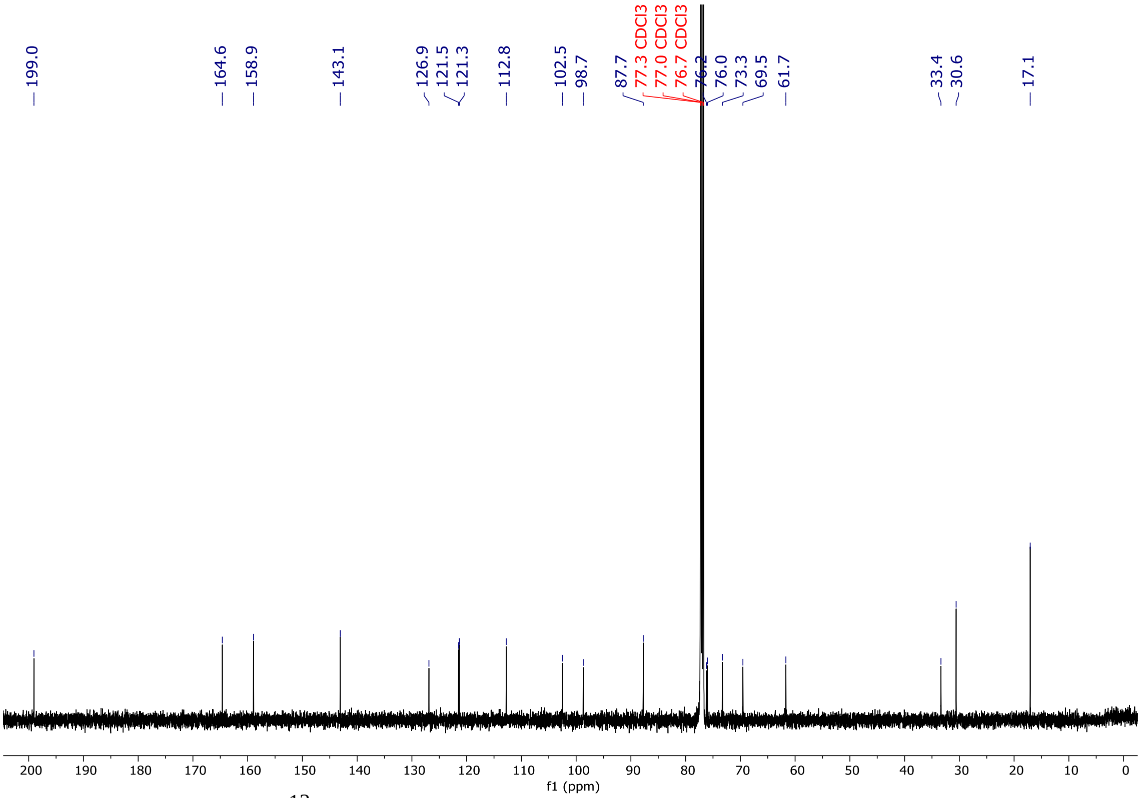

Figure S23. ^{13}C-NMR spectrum of compound **18** (CDCl3, 125 MHz)

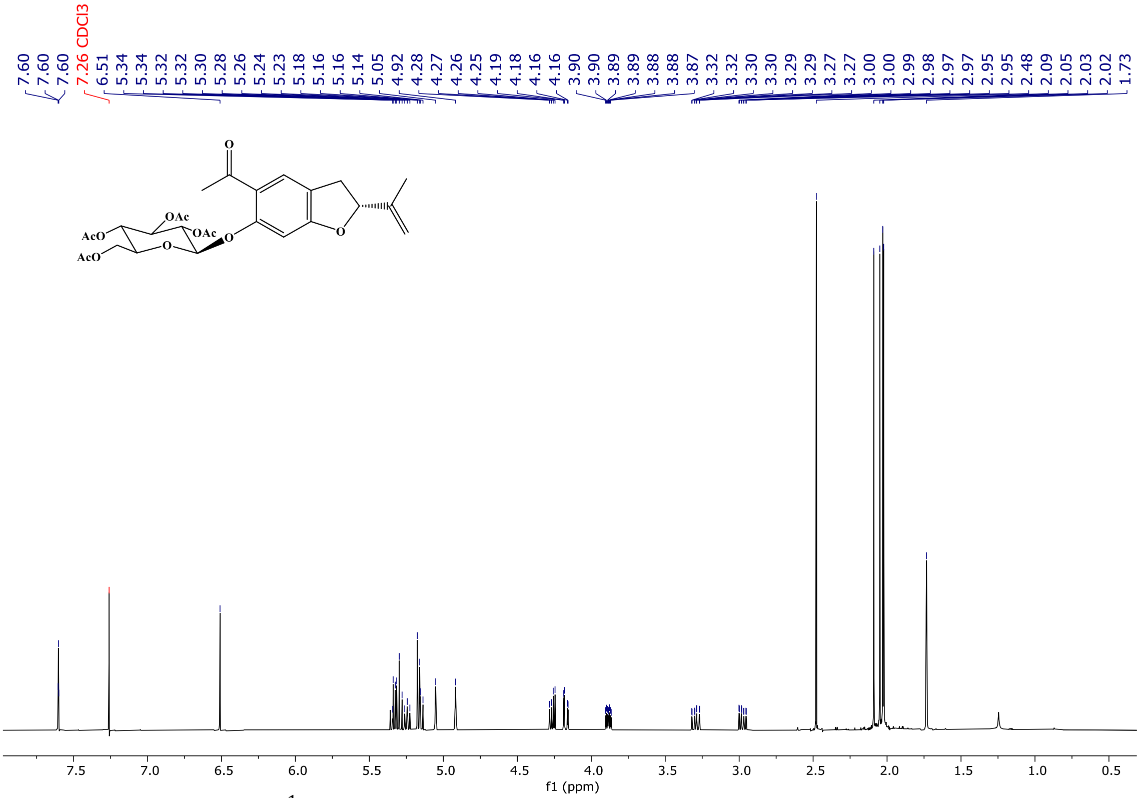

Figure S24. ^{1}H-NMR spectrum of compound **18a** ($CDCl_3$, 500 MHz)

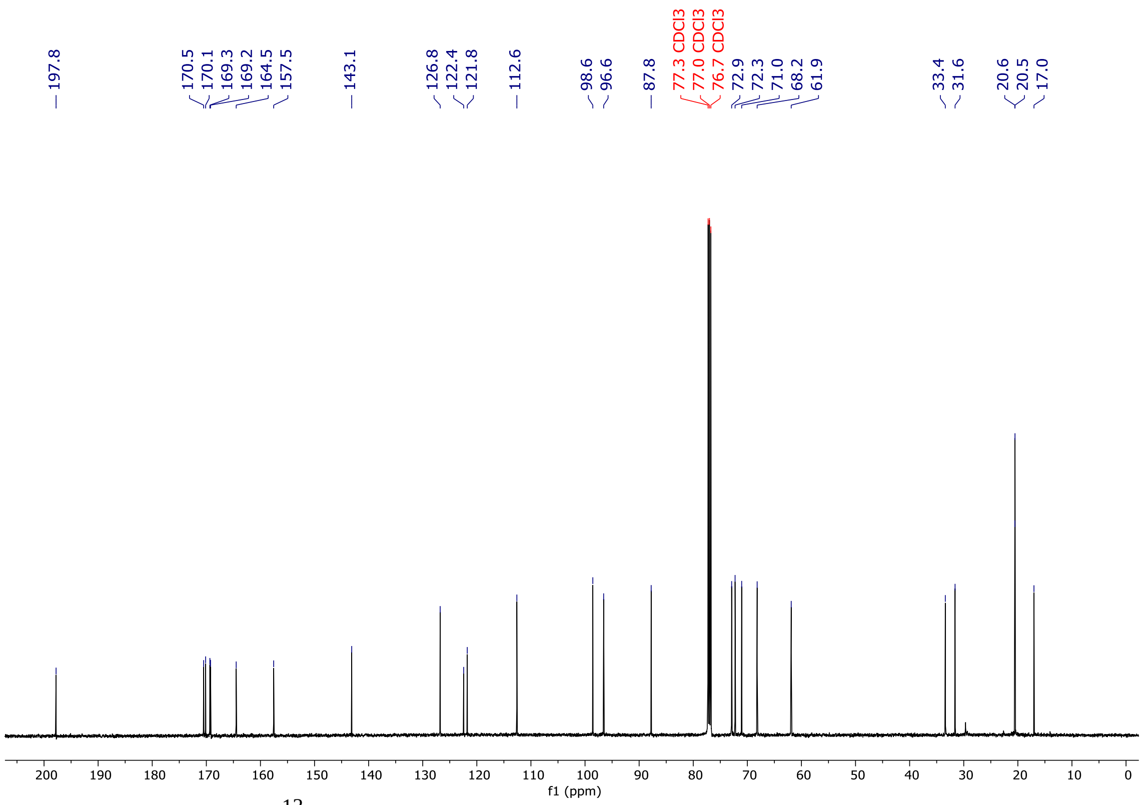

Figure S25. ^{13}C-NMR spectrum of compound **18a** (CDCl3, 125 MHz)

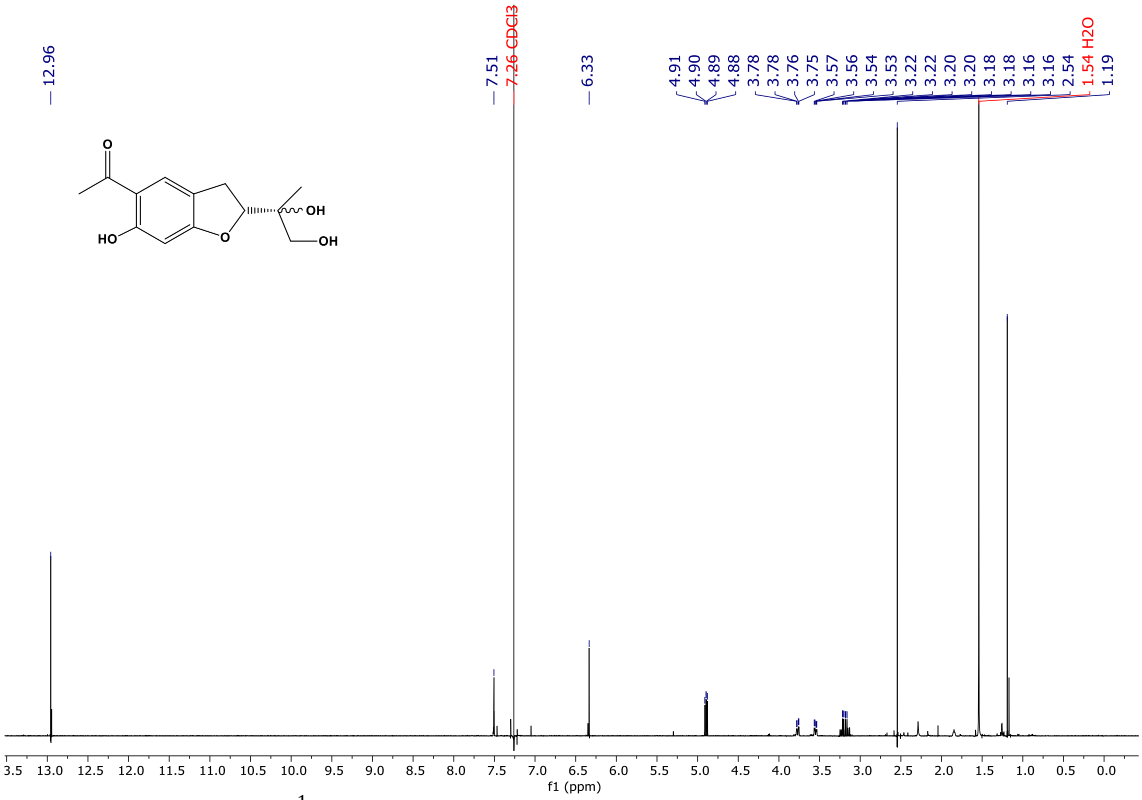

Figure S26. ^{1}H-NMR spectrum of compound **19** ($CDCl_3$, 500 MHz)

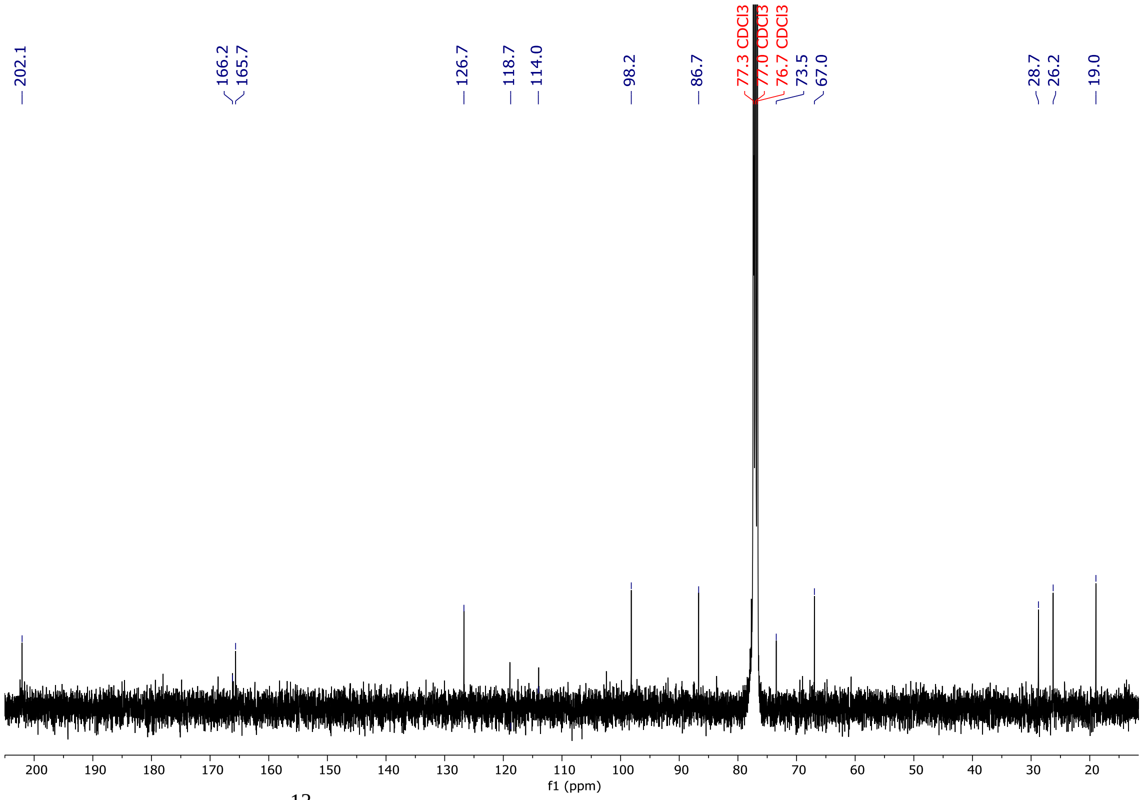

Figure S27. ^{13}C-NMR spectrum of compound **19** (CDCl3, 125 MHz)
